# Comprehensive Phytochemical Analysis and Bioactivity Evaluation of *Padina boergesenii*: Unveiling Its Prospects as a Promising Cosmetic Component

**DOI:** 10.3390/md21070385

**Published:** 2023-06-29

**Authors:** Haresh S. Kalasariya, Leonel Pereira, Nikunj B. Patel

**Affiliations:** 1 Centre for Natural Products Discovery, School of Pharmacy and Biomolecular Sciences, Liverpool John Moores University, Byrom Street, Liverpool L3 3AF, UK; hareshahir22@gmail.com; 2 MARE–Marine and Environmental Sciences Centre/ARNET–Aquatic Research Network, Department of Life Sciences, University of Coimbra, Calçada Martim de Freitas, 3000-456 Coimbra, Portugal; 3 Microbiology Department, Sankalchand Patel University, Visnagar 384315, Gujarat, India; nbpatel.fsh@spu.ac.in

**Keywords:** *Padina boergesenii*, marine macroalgae, cosmetics, HRLCMS QTOF, GCMS, FTIR

## Abstract

Marine macroalgae, such as *Padina boergesenii*, are gaining recognition in the cosmetics industry as valuable sources of natural bioactive compounds. This study aimed to investigate the biochemical profile of *P. boergesenii* and evaluate its potential as a cosmetic ingredient. Fourier-transform infrared (FTIR), gas chromatography–mass spectrometry (GCMS), and high-resolution liquid chromatography–mass spectrometry quadrupole time-of-flight (HRLCMS QTOF) analyses were employed to assess the functional groups, phycocompounds, and beneficial compounds present in *P. boergesenii*. Pigment estimation, total phenol and protein content determination, DPPH antioxidant analysis, and tyrosinase inhibition assay were conducted to evaluate the extracts’ ability to counteract oxidative stress and address hyperpigmentation concerns. Elemental composition and amino acid quantification were determined using inductively coupled plasma atomic emission spectrometry (ICP-AES) and HRLCMS, respectively. FTIR spectroscopy confirmed diverse functional groups, including halo compounds, alcohols, esters, amines, and acids. GCMS analysis identified moisturizing, conditioning, and anti-aging compounds such as long-chain fatty alcohols, fatty esters, fatty acids, and hydrocarbon derivatives. HRLCMS QTOF analysis revealed phenolic compounds, fatty acid derivatives, peptides, terpenoids, and amino acids with antioxidant, anti-inflammatory, and skin-nourishing properties. Elemental analysis indicated varying concentrations of elements, with silicon (Si) being the most abundant and copper (Cu) being the least abundant. The total phenol content was 86.50 µg/mL, suggesting the presence of antioxidants. The total protein content was 113.72 µg/mL, indicating nourishing and rejuvenating effects. The ethanolic extract exhibited an IC_50_ value of 36.75 μg/mL in the DPPH assay, indicating significant antioxidant activity. The methanolic extract showed an IC_50_ value of 42.784 μg/mL. Furthermore, *P. boergesenii* extracts demonstrated 62.14% inhibition of tyrosinase activity. This comprehensive analysis underscores the potential of *P. boergesenii* as an effective cosmetic ingredient for enhancing skin health. Given the increasing use of seaweed-based bioactive components in cosmetics, further exploration of *P. boergesenii*’s potential in the cosmetics industry is warranted to leverage its valuable properties.

## 1. Introduction

Cosmeceutical products, as a fusion of cosmetic and pharmaceutical elements, harness the power of biologically active ingredients to enhance skin health [1]. With the modern lifestyle and increasing beauty concerns, the global beauty care industry is experiencing rapid growth. However, the widespread use of synthetic cosmetics and ingredients has raised concerns regarding their effectiveness and potential harm to the skin. For instance, hydroxybenzoic acid esters (parabens), widely utilized in cosmetic formulations, have been linked to harmful effects on the skin, including an increased incidence of breast cancer and malignant melanoma [2]. Phthalates, found in numerous cosmetic products, have demonstrated the ability to induce DNA modifications and damage in human sperm cells [3]. Moreover, animal studies have revealed detrimental effects such as decreased sperm counts and congenital disabilities in offspring associated with exposure to certain synthetic chemicals [4]. Consequently, consumers are increasingly opting for natural cosmetic products, leading to the development of a wide array of skin cosmetic formulations [5].

Seaweeds, also known as macroalgae, are eukaryotic, multicellular, macroscopic, marine photosynthetic organisms that are ubiquitously distributed along coastlines, ranging from tropical to polar regions. They primarily inhabit intertidal and subtidal regions of coastal areas [6]. Macroalgae are classified into three major types: brown algae (Ochrophyta phylum), red algae (Rhodophyta phylum), and green algae (Chlorophyta phylum). While green and red algae belong to the Plantae kingdom, brown algae belong to the Chromista kingdom [7]. Marine macroalgae represent a rich reservoir of structurally diverse biologically active constituents. Notably, algae exhibit 10-fold greater phycocompound diversity compared to terrestrial plants. These biologically active constituents found in marine macroalgae possess multifaceted activities that make them suitable for incorporation into cosmetic formulations. Their valuable bioactive compounds include carbohydrates, proteins, amino acids, lipids, fatty acids, phenolic compounds, pigments, vitamins, and minerals [8,9]. The broad range of applications for marine macroalgae is derived from these inherent bioactive compounds and their potential bioactivities, making them highly sought after in the development of cosmetic formulations.

*Padina boergesenii*—a distinct, small brown alga—stands out among the marine macroalgae. This species features rounded fronds that reach a length and diameter of 4 to 6 cm (1.6 to 2.4 in). Its fronds are thin, leafy, and flat, exhibiting narrow or wide lobes. Typically, *P. boergesenii* displays a pale brown or tan coloration, with a moderate amount of calcification on the underside [10,11]. The fronds are generally two or three cells thick, with the bases commonly being three cells thick. The surface of the fronds is adorned with numerous short hairs, contributing to their matted appearance [12]. The choice of *Padina boergesenii* as the focus of this study stems from its unique characteristics and potential as a valuable natural cosmetic ingredient. The aforementioned characteristics of *P. boergesenii*, combined with its abundance in coastal areas, make it an intriguing candidate for exploration in the cosmetics industry. Furthermore, previous research studies have highlighted the extensive range of biological activities exhibited by seaweed-derived active molecules, offering a variety of skin benefits.

Previous studies have demonstrated the extensive range of biological activities exhibited by seaweed-derived active molecules, which offer a variety of skin benefits. The objective of this study was to investigate the biochemical profile of *P. boergesenii*—a marine macroalga—employing various analytical techniques, including FTIR, GCMS, HRLCMS QTOF, pigment estimation, total phenol content, total protein content, antioxidant analysis, and tyrosinase inhibition assay. This study aimed to assess the potential of *P. boergesenii* as a natural cosmetic ingredient by exploring its bioactive constituents, elemental composition, amino acid composition, and its ability to improve skin health. The findings of this study contribute to our understanding of *P. boergesenii*’s suitability for incorporation into seaweed-based cosmetic products, further fueling research and development in the cosmetics industry. By elucidating the biochemical composition and evaluating its bioactive properties, this investigation aims to unlock the potential of *P. boergesenii* as a natural and sustainable source for enhancing skin health and shedding light on it as a potential cosmetic ingredient.

## 2. Results and Discussion

### 2.1. Collection of Brown Alga P. boergesenii

**Figure 1:**
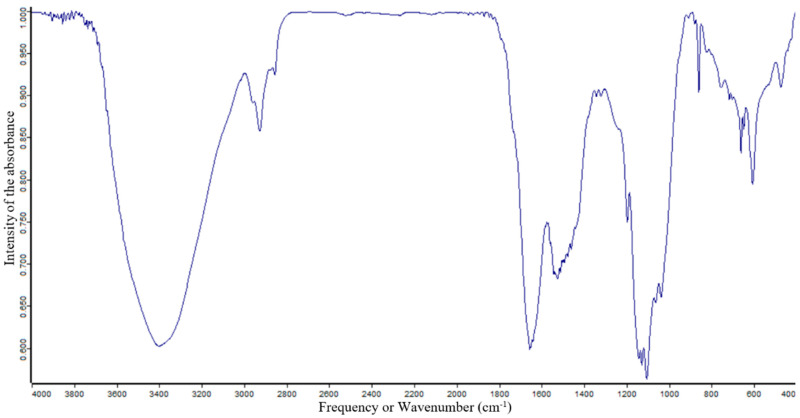
FTIR spectrum of *Padina boergesenii* using the KBr pellet method.

Among the macroalgal species surveyed in the coastal region under investigation, *Padina boergesenii* was found to exhibit significant prominence during both the winter and summer seasons, displaying a higher degree of persistence compared to other co-occurring algal species. This species demonstrates optimized growth and survival within specific environmental conditions. Notably, *P. boergesenii* predominantly colonizes intertidal pools situated in the upper–middle region of the coast, with a propensity for attachment to sandy or muddy benthic substrates, and occasional formation of aggregations on the water surface. The study area, located at Beyt Dwarka sea coast (Figure 1) along the western coast of Gujarat, provides an ideal habitat for *P. boergesenii*, thereby rendering it a suitable location for the present investigation. The collection of *P. boergesenii* samples involved targeted sampling from the intertidal zone during low tidal conditions to ensure ease of accessibility. Specifically, the intertidal area was sampled under controlled environmental parameters, including a relative humidity level of 55%, wind velocity measuring 11 km/h, ambient temperature maintained at 27 °C, and the absence of precipitation. By maintaining controlled environmental conditions during the sample collection process, we aimed to minimize the potential influence of external factors on the observed results. This approach allows for a more focused evaluation of the inherent biochemical profile of *P. boergesenii* and provides a foundation for understanding its potential as a cosmetic ingredient.

The taxonomic details of *P. boergesenii* can be found at the following link: https://www.algaebase.org/search/species/detail/?species_id=1312 [13].

### 2.2. Functional Group Analysis of P. boergesenii Using FTIR Spectroscopy

The functional groups of the active compounds in *P. boergesenii* were characterized using FTIR analysis, which involved assessing the peak values, frequencies, and intensities, and assigning bands to specific functional groups. The FTIR spectrum of the studied sample is depicted in Figure 1, and the corresponding values for selected algae are presented in Table 1. In the FTIR analysis of *Padina boergesenii*, prominent bands were observed at frequencies of 604, 659, 751, 1038, 1099, 1126, 1196, 1320, 1343, 1521, 1648, 1656, 1873, 2859, 2925, 2959, 3404, and 3686 cm^−1^. These bands were attributed to specific functional groups, including C-O, C-H, S=O, C-Br, C-Cl, C=C, S=O, N-H, C-F, C=N, C-N, and O-H, indicating the presence of halo compounds, 1,2-disubstituted compounds, monosubstituted compounds, sulfoxides, fluoro compounds, amines, aliphatic ethers, secondary alcohols, tertiary alcohols, esters, aromatic amines, phenols, sulfonates, sulfonamides, sulfonic acids, sulfones, imines/oximes, alkenes, conjugated alkenes, carboxylic acids, amine salts, and alkanes within the *P. boergesenii* extract.

These identified functional groups play crucial roles in providing various skin benefits and can be utilized in cosmetic formulations. For instance, alcohols, such as secondary and tertiary alcohols, are known for their potential moisturizing and emollient properties, contributing to improved skin hydration and texture [14,15]. Esters, carboxylic acids, and phenols may have antioxidant and anti-inflammatory effects, which can support overall skin health and combat signs of aging [16,17]. Amines and sulfonic acids might exhibit antimicrobial properties, helping to protect the skin against bacterial and fungal infections [18,19]. Furthermore, the presence of halo compounds may impart potential photoprotective effects, aiding in shielding the skin from harmful UV radiation [20]. The FTIR analysis provides valuable insights into the functional groups present in *P. boergesenii*, highlighting their significance in skincare and cosmetic applications. Further exploration and understanding of the specific contributions of these functional groups can lead to the development of innovative and effective cosmetic formulations targeting moisturization, anti-aging, photoprotection, and antimicrobial properties.

### 2.3. Characterization of Ethanolic Extracts of P. boergesenii Using GCMS

**Figure 2:**
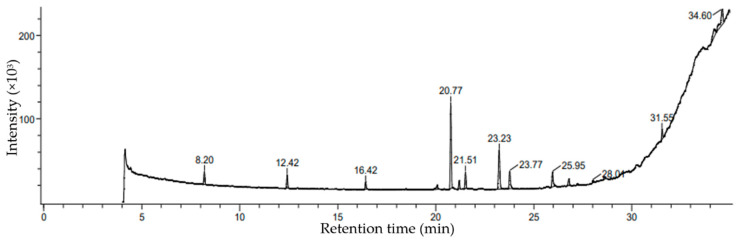
The gas chromatogram of ethanolic extract from *Padina boergesenii* marine macroalga.

Based on percentage peak area, retention time (in min), molecular formula, and molecular weight, different compounds were characterized in the ethanolic extracts of the selected marine macroalga. The gas chromatogram of the selected macroalga, with different peaks, is illustrated in Figure 2. The chemical information of different compounds obtained in the GCMS analysis is tabulated in Table 2. 

The analysis of the brown alga *P. boergesenii* revealed the presence of a variety of compounds with potential cosmetic benefits. Among these compounds, a long-chain fatty alcohol (3,7,11,15-tetramethylhexadec-2-en-1-ol) stood out, with the highest peak area (28.64%) and a retention time of 20.77 min. This compound has shown promise in providing skincare benefits (mainly skin moisturization), although further studies are needed to fully understand its specific effects [21]. In addition to 3,7,11,15-tetramethylhexadec-2-en-1-ol, other noteworthy compounds were identified in the ethanolic extracts of *P. boergesenii*. Hexadecanoic acid, ethyl ester; 17-octadecen-1-ol acetate; and oxalic acid, allyl nonyl ester were found to be fatty esters, which are known for their potential moisturizing and conditioning properties in skincare products [22,23]. Further compounds identified included fatty acyls, such as decane, 6-ethyl-2-methyl- and octadecane, 3-ethyl-5-(2-ethylbutyl)-, which may contribute as surfactants in skincare products [24,25]. Fatty acid derivatives, such as 2-monolinolenin, 2TMS derivatives, and benzyl (6Z,9Z,12Z)-6,9,12-octadecatrienoate hold potential for their ability to provide antioxidant and anti-inflammatory effects on the skin, contributing to overall skin health and anti-aging properties [26,27,28]. Hydrocarbon derivatives, including cyclopentanol, 2-methyl-, trans-, and cyclooctane acetic acid, 2-oxo-, may have unique properties that can contribute to the sensory aspects and formulation of cosmetic products [29,30]. Among the compounds screened, pentadecanoic acid, tripropyl silyl ester, bispalmitic acid 3-methyl-1,2-butanediyl, cyclodecanol, 9-(2-Oxiranyl)-1-nonanol, phthalic acid, 6-ethyl-3-octyl butyl ester, and 3,7,11,15-tetramethylhexadec-2-en-1-ol (a diterpene) exhibited potential skincare benefits, including moisturization, anti-aging effects, and overall skin health support [31,32,33].

In addition, the analysis of *P. boergesenii* revealed several other compounds with potential cosmetic benefits. Pentadecanoic acid, tripropyl silyl ester—a straight-chain fatty acid derivative—has shown promising properties in terms of moisturization and skin conditioning [34,35,36]. Its presence suggests that it could contribute to the emollient and barrier-enhancing effects of skincare formulations. Bispalmitic acid 3-methyl-1,2-butanediyl was another compound of palmitic acid that was identified and may possess unique characteristics that contribute to the overall health and texture improvement of skin [37,38]. Cyclodecanol and 9-(2-oxiranyl)-1-nonanol—two fatty alcohols found in *P. boergesenii*—have been associated with emollient properties and the potential to improve the texture and feel of cosmetic products on the skin [39,40]. These compounds may contribute to the overall sensory experience and moisturizing effects of skincare formulations. Phthalic acid, 6-ethyl-3-octyl butyl ester—a carboxylic acid derivative—may offer benefits such as improved skin penetration and moisturization [41,42]. Furthermore, the diterpene compound 3,7,11,15-tetramethylhexadec-2-en-1-ol, in addition to its antioxidant potential, may exhibit unique characteristics that contribute to skin health and anti-aging effects [43,44]. Its inclusion in skincare formulations could help protect against oxidative stress and UV-induced damage. The presence of these compounds in *P. boergesenii* highlights its potential as a rich source of diverse bioactive compounds for cosmetic applications. By harnessing the properties of these compounds, skincare formulations can be developed to offer moisturizing, barrier-enhancing, emollient, and anti-aging benefits. Further research is necessary to fully explore the mechanisms of action and optimize the utilization of these compounds in cosmetic formulations.

### 2.4. Comprehensive Profiling of Methanol-Extracted Phycocompounds in P. boergesenii Using GCMS Analysis 

**Figure 3:**
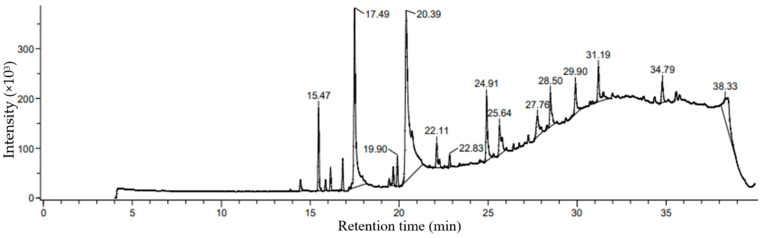
Gas chromatographic analysis of fatty acids and other bioactive compounds in methanolic extracts of the marine macroalga *P. boergesenii*.

Based on percentage peak area, retention time (in minutes), molecular formula, and molecular weight, different compounds were characterized in methanolic extracts of selected marine macroalgal species. Gas chromatograms of the selected macroalga with different peaks are illustrated in Figure 3. The chemical information of the different compounds obtained in the GCMS analysis is tabulated in Table 3.

The methanolic extract of *P. boergesenii* was subjected to GCMS analysis after the derivatization of fatty acids using the fatty acid methyl ester (FAME) method. This derivatization step allowed for enhanced stability and volatility of the fatty acids, enabling accurate quantification and identification during the GCMS analysis. The GCMS analysis revealed several compounds present in the methanolic extract of *P. boergesenii*, each with its own potential role in skincare and cosmetics. Among the identified compounds, 9-octadecenal exhibited the highest percentage peak area (34.79%) at a retention time of 2.36 min. This compound belongs to the class of fatty aldehydes and is known for its pleasant aroma, which could be utilized in cosmetic products for fragrance purposes [45]. In terms of fatty acids, the analysis identified hexadecanoic acid methyl ester, palmitic acid, and palmitic acid vinyl ester as the saturated fatty acids present in *P. boergesenii*. These fatty acids provide moisturizing and emollient effects, helping to hydrate and soften the skin [46,47,48]. Additionally, the unsaturated fatty acids cis-9-octadecenoic acid and 9-dodecenoic acid methyl ester [E]-, along with alkynyl stearic acid, were also detected. Unsaturated fatty acids are known for their skin-nourishing properties and can contribute to maintaining the skin’s barrier function [49,50,51]. Other noteworthy compounds found in *P. boergesenii* included hexahydrofarnesol, a fatty alcohol that can provide hydration and soothing benefits to the skin [52]. Fatty aldehydes, such as 9-octadecenal and (z)-9,17-octadecadienal, may contribute to the sensory experience and aroma of cosmetic products [53]. 

Phytosterols like 29-methyliso fucosterol are bioactive compounds with potential antioxidant and anti-inflammatory effects [54]. Terpenes such as phytol and patchoulol are known for their antimicrobial properties and can be beneficial in formulations targeting acne or other skin concerns [55,56]. Cyclic organic compounds like 1,2,4-trioxolane, 3,5-dipropyl- can have antioxidant and skin-soothing effects [57]. Hydrocarbon derivatives like 1,1’-bicyclopentyl, 2-hexadecyl-, and 1,4-eicosadiene offer potential benefits as skin-conditioning agents, providing moisture retention and improving the overall appearance of the skin [58,59]. These individual compounds found in *P. boergesenii* present various opportunities for formulators to develop skincare products with targeted benefits. The saturated and unsaturated fatty acids contribute to moisturization and skin barrier support, while the fatty aldehydes, fatty alcohols, and other compounds offer sensory enhancements and specific skincare properties [60,61]. By harnessing the potential of these compounds, cosmetic formulations can be tailored to address specific skin concerns and provide an enjoyable and effective skincare experience. Further research and exploration of these compounds will unlock their full potential and expand their application in the cosmetics industry.

### 2.5. Comprehensive Characterization of Phycocompounds in P. boergesenii Using HRLCMS QTOF

**Figure 4:**
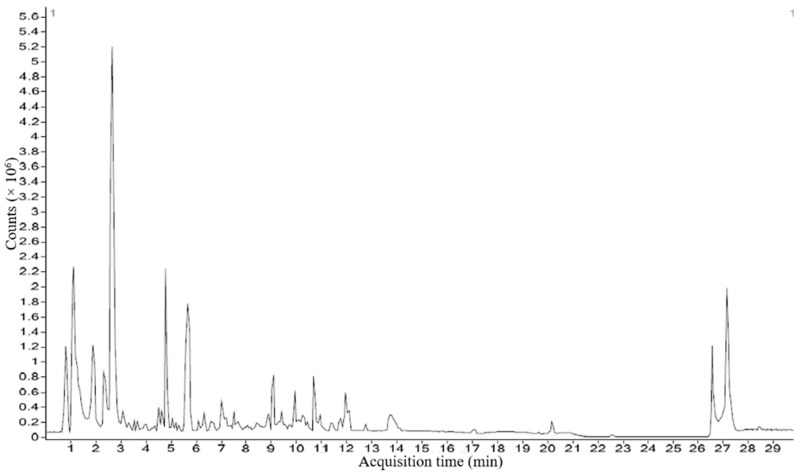
Liquid chromatogram (LC) for phycocompounds’ characterization from the selected macroalga *Padina boergesenii* by HRLCMS QTOF. X-axis: acquisition time (min); Y-axis: counts (×10^6^).

An analysis of methanolic extracts from the selected marine macroalga revealed different types of phycocompounds with different values of percentage peak area and retention time. In the characterization study, the liquid chromatogram (LC) for the screened alga obtained by HRLCMS analysis is illustrated in Figure 4. The different types of phycocompounds obtained for this brown alga, along with their details, are tabulated in Table 4. Unknown compounds were identified and characterized by comparing the obtained values of unknown compounds with standard available data in the library.

In *P. boergesenii*, the compound 2,6-dimethoxy-4-(1-propenyl)phenol—a phenolic compound—exhibits potent antioxidant activity, protecting the skin from oxidative stress and combating signs of aging [62,63]. Fatty acid derivatives, such as 8-amino caprylic acid, N-linoleoyl taurine, and bolekic acid, offer moisturizing and nourishing effects on the skin. These compounds can help maintain skin hydration, improve skin texture, and support overall skin health [64,65]. Peptide molecules, including L-NIO, Lys Gly, Pro Pro His, Gln Phe Lys, Tyr Ile Pro, S-Decyl GSH, Lys Met Lys, and N-formyl-norleucyl-leucylphenylalanyl-methylester, possess diverse skincare benefits. They may stimulate collagen synthesis, improve skin elasticity, and contribute to anti-aging effects [66,67,68]. These peptides can be incorporated into skincare formulations targeting wrinkle reduction and improving skin firmness [69,70]. Phycocompounds such as Ni-azirinin—a carbohydrate derivative—hold potential for moisturization and skin conditioning [71]. Cryptopleurine, an alkaloid, may exhibit antimicrobial and anti-inflammatory effects, making it useful in skincare products targeting acne or skin inflammation [72]. The presence of LG 100268, a member of the vitamin B complex, suggests potential benefits in supporting overall skin health, cellular functions, and rejuvenation [73]. Terpenoids, like phytol and patchoulol, offer potential antioxidant and antimicrobial properties, which can help protect the skin from environmental stressors and maintain a healthy complexion [74,75]. By harnessing the potential benefits of these individual compounds, skincare formulations can be developed to target specific skin concerns, such as aging, dryness, inflammation, and microbial imbalances. Further research and studies are needed to fully understand the mechanisms of action and determine optimal concentrations for these compounds in cosmetic applications.

### 2.6. Quantification of Amino Acids in P. boergesenii Using HRLCMS

**Figure 5:**
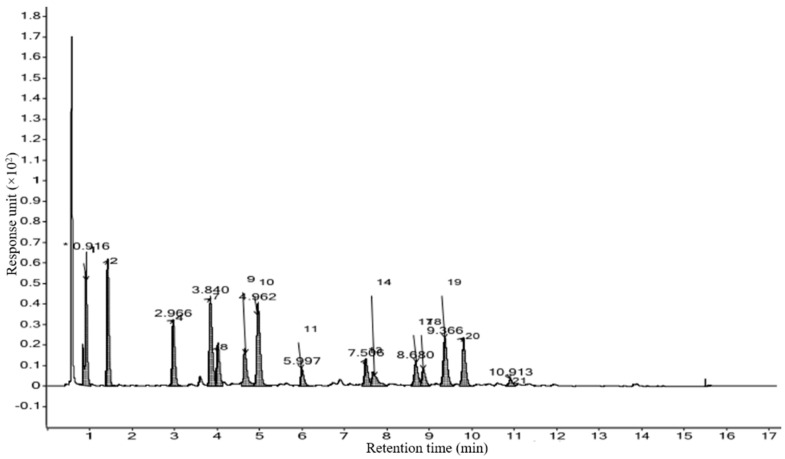
Liquid chromatogram (LC) for different amino acids from the selected macroalga *Padina boergesenii* by HRLCMS QTOF.

In total, 21 different types of amino acids were measured in the chosen marine macroalga by the HRLCMS QTOF technique. The liquid chromatogram (LC) showing the peaks of different amino acids in *Padina boergesenii* alga is illustrated in Figure 5, and its contents are tabulated in Table 5 (Calibration curves for amino acid standards attached in Sheet S1 of Supplementary File). In the present study, *P. boergesenii* revealed a good amount of different amino acids Likewise, *P. boergesenii* showed more than 500 nmol/mL contents of Asp, Glu, Ala, Gly, Leu, Hyp, Ser, and Thr. Contents of amino acids were found in the following descending order (in nmol/mL) in *P. boergesenii*: Asp > Glu > Ala > Gly > Leu > Hyp > Ser > Thr > Arg > Phe > Val > Lys > Met > Ile > Tyr. It should be noted that the characterization of these amino acids primarily focused on their identification and quantification, without specific differentiation between free and bound forms. The analysis aimed to provide a comprehensive profile of the amino acid composition in *P. boergesenii*. Further investigations, specifically targeting the differentiation of free and bound amino acids, would be valuable to gain a deeper understanding of the nature and distribution of amino acids in this macroalga.

Many previous studies have suggested skin-beneficial actions of amino acids, such as antioxidant, anti-inflammatory, photoprotective, anti-wrinkle, moisturization, anti-elastase, and other skin-health-promoting benefits [76]. Among different amino acids, proline, glycine, and leucine are found abundantly in collagen, and lysine, histidine, and arginine are the three most common useful amino acids for skincare preparations. Likewise, the combination of arginine and lysine is noted for its skin-healing benefits, while the combination of leucine and proline has been reported for repairing skin wrinkles [77]. Moreover, lysine plays an important functional role in elastin and collagen [78], and it has proven beneficial for preventing acne and cold sores. Choi et al. [79] showed the role of tripeptides containing histidine and lysine for skin moisturization in skincare formulations. The synthesis of dermal collagen was considerably stimulated by leucine, isoleucine, arginine, glycine, proline, and valine in hairless mice against the damaging effects of UV [80]. Mixing leucine with glycine and proline was found to be useful for the repair of skin wrinkles [81]. On the faces of Japanese women, skin elasticity in crow’s feet lines was found to have been improved after topical treatment with a proline derivative [81]. As a moisturizing agent, alanine and serine were found to be useful in skincare product preparation and played a greater role in water retention in the stratum corneum layers. Yamane et al. [82] showed the role of isoleucine and leucine in the collagen synthesis of the skin. Patients with facial atopic eczema underwent treatment using a specialized emollient cream formula that incorporated isoleucine-containing ceramides [83]. Aromatic amino acids, tyrosine, and phenylalanine play an important role as precursors in melanin synthesis. It is the main skin cutaneous pigment that provides protection against harmful sunlight containing UV rays and protects against DNA damage and skin cancers [84]. Moreover, tyrosine and phenylalanine were found to play an important role in the melanin synthesis process [85]. A sulfur-containing amino acid, methionine can also provide anti-acne, anti-inflammatory, and antioxidant benefits [86]. Furthermore, serine and threonine remain useful for moisture regulation in the stratum corneum. Moreover, proline and glycine amino acids are not only present as components in collagen but also work as regulators of its synthesis [87].

### 2.7. Elemental Analysis of P. boergesenii Using the ICP-AES Technique

Marine macroalgae are highly rich in different mineral elements. These marine-algae-derived minerals can be applied for skin cosmetic benefits. Among the measured minerals, silicon, potassium, calcium, magnesium, and iron have been found in major amounts in studied macroalgae species. The present study suggested that silicon (Si) was found to be the most abundant, whereas copper (Cu) was the least abundant in the selected sample. In total, 10 elements, along with their measured concentrations (%) in the alga, are tabulated in Table 6. The percentages of the minerals in the selected macroalga were as follows, in decreasing order (in %): silicon > calcium > potassium > magnesium > iron > sodium > boron > zinc > copper. Based on the phytochemical analysis conducted in this study, *P. boergesenii* was found to contain a range of minerals, including silicon, potassium, calcium, magnesium, and iron, among others. These minerals are known to have beneficial effects on the skin and can contribute to skin health and cosmetic benefits. However, it is important to consider the safety of using *P. boergesenii* in cosmetic formulations. As the focus of this study was primarily on the phycochemical analysis, a thorough safety assessment was not conducted. To evaluate the safety of *P. boergesenii* for cosmetic purposes, further research is required. Safety assessments typically involve a comprehensive analysis of potential irritancy, sensitization, and cytotoxicity, among other parameters, to ensure the safe use of a natural ingredient in cosmetic products. While the phytochemical profile of *P. boergesenii* suggests its potential benefits for cosmetic applications, it is crucial to conduct specific safety evaluations to determine its suitability for use in skincare formulations. Future studies should encompass rigorous safety assessments to address any potential concerns and ensure the overall safety of *P. boergesenii* in cosmetic applications.

Many studies have reported the beneficial actions of minerals in promoting skin health. Calcium plays an important role in hemostasis as well as in key regulation of epithelialization, which is significantly noteworthy for the differentiation of basal keratinocytes to corneocytes [88]. Moreover, calcium (Ca^++^) was found to be a key modulator in keratinocytes’ locomotion and a promoter of wound healing in an in vivo study. For the maintenance of cell membrane potential, sodium and potassium demonstrated beneficial roles at the cellular and extracellular levels as electrolytes and osmolytes. Regarding skin benefits, formulations prepared from Dead Sea salts containing magnesium were found to be useful for skin nourishment [89]. Denda et al. [90] reported the skin-repairing role of calcium and magnesium salts. In the experiment, it was observed that topical treatment with 5% magnesium chloride (MgCl_2_) before exposure to UVB radiation resulted in a reduction in the number of Langerhans cells in the epidermis and a decrease in antigen-presenting activity in the skin [91]. However, zinc was found to be safer and more effective in many topical applications. According to the report of the FDA of the United States, three zinc-containing compounds (zinc oxide, zinc carbonate, and zinc acetate) showed safer and more effective application as topical skin protectants [92]. Some other studies have suggested the important wound-healing role of zinc in tissue. Zinc oxide (ZnO) demonstrated a sunscreen-like protective role, as well as effectiveness for the treatment of skin rashes, pruritus, psoriasis, ringworm, impetigo, ulcers, and eczema [93]. Another important element, iron (Fe), was found to be most abundant in trace metals with various skin benefits. Most importantly, it showed major action in skin-relevant procollagen-proline dioxygenases [94]. More interestingly, iron (Fe) was helpful against UV-induced damaging effects, with a higher amount of iron being expressed in UV-exposed skin (53.0 ppm) than in unexposed skin (17.8 ppm) [95]. Moreover, copper (Cu) showed an effective role in the stimulation of collagen formation and improvement of skin health. In beauty products, copper peptides have been found to relax skin irritation, improve skin elasticity and skin firmness, heal photodamaged skin, decrease fine lines, and treat skin wrinkles, among other benefits [96].

### 2.8. Determination of Pigment Content in Padina boergesenii

#### 2.8.1. Quantification of Chlorophylls, Carotenoids, Fucoxanthin, Phycoerythrin, and Phycocyanin

The pigment content of *P. boergesenii* was analyzed using various solvents to extract different classes of pigments. The extraction process was carried out following established protocols and in triplicate to ensure accuracy. In the 100% methanolic extract of *P. boergesenii*, the concentrations of chlorophyll a, chlorophyll b, chlorophyll c, and carotenoids were found to be 4.15 µg/mL, 9.16 µg/mL, 2.05 µg/mL, and 0.93 µg/mL, respectively. On the other hand, in the 100% ethanolic extract, the concentrations of chlorophyll a, chlorophyll b, chlorophyll c, and chlorophyll d were determined to be 3.55 µg/mL, 2.53 µg/mL, 8.15 µg/mL, and 2.18 µg/mL, respectively. The analysis of pigment content in *P. boergesenii* is of particular interest due to the potential cosmetic benefits associated with these pigments. Pigments derived from natural sources, such as algae, have gained attention in the cosmetic industry for their various functional properties and aesthetic appeal.

Several related studies have demonstrated the importance of algal pigments in cosmetic applications. For instance, chlorophylls, which are major photosynthetic pigments found in algae, have been recognized for their antioxidant and anti-inflammatory properties. These properties are beneficial in skincare products, as they help protect the skin from oxidative stress and contribute to a healthier and more youthful appearance [97]. Carotenoids, another class of pigments found in algae, have shown significant potential in cosmetic formulations. These pigments are known for their antioxidant properties and their ability to scavenge free radicals, which can contribute to premature aging and skin damage. Carotenoids, such as β-carotene and astaxanthin, have been extensively studied for their photoprotective effects against UV-induced skin damage, including the prevention of wrinkle formation and improvement of skin elasticity [98,99]. Fucoxanthin, a brown pigment specific to brown algae, has also attracted attention in the cosmetics industry. It has been reported to have anti-aging and skin-lightening properties. Fucoxanthin exhibits antioxidant activity and inhibits the activity of tyrosinase (an enzyme involved in melanin synthesis), which makes it a potential ingredient in skin-whitening and -brightening products [100]. Moreover, the red pigments phycoerythrin and phycocyanin, extracted from certain red algae species, have been investigated for their cosmetic applications. These pigments possess strong antioxidant and anti-inflammatory properties, making them valuable for skincare products targeted at reducing skin redness and inflammation, and providing overall skin protection [101]. By determining the pigment contents in *P. boergesenii*, this study contributes to our understanding of the potential cosmetic benefits associated with these algae. The presence of chlorophylls, carotenoids, fucoxanthin, phycoerythrin, and phycocyanin in the algal extract suggests that *P. boergesenii* could be a promising natural source for cosmetic formulations aiming to harness the antioxidant, anti-aging, skin-lightening, and anti-inflammatory properties of these pigments.

Using the same method with a DMSO:water (4:1, *v*/*v*) solvent mixture, no fucoxanthin was detected in the *P. boergesenii* sample. Fucoxanthin is a specific brown pigment typically found in brown algal species and is known for its various beneficial properties, including antioxidant and skin-lightening effects. However, the absence of fucoxanthin in the analyzed sample suggests that *P. boergesenii* may not contain significant levels of this particular pigment. It is important to consider that the presence or absence of fucoxanthin can vary between different algal species and may be influenced by various factors such as growth conditions, geographical location, and extraction methods [102]. Further investigation or alternative extraction techniques may be necessary to determine the presence of fucoxanthin in *P. boergesenii* or to explore other potential sources of this pigment.

Using phosphate buffer (pH = 6.8) as the solvent, the pigment analysis of *P. boergesenii* revealed the presence of phycoerythrin at a concentration of 26.15 µg/mL and of phycocyanin at a concentration of 39.90 µg/mL. Phycoerythrin and phycocyanin are known as accessory pigments, commonly found in various species of red algae (Rhodophyta). These pigments play important roles in photosynthesis, light absorption, and energy transfer within the algal cells. Additionally, phycoerythrin and phycocyanin have shown potential applications in the cosmetics industry due to their antioxidant properties and their ability to scavenge free radicals, which contribute to skin aging and damage. The presence of these pigments in *P. boergesenii* suggests its potential as a natural source of phycoerythrin and phycocyanin for cosmetic formulations aimed at promoting skin health and protection against oxidative stress.

#### 2.8.2. Estimation of Chlorophylls and Lycopene

By employing an acetone–hexane mixture (2:3) as the extraction solvent, the estimation of chlorophylls and lycopene in *P. boergesenii* was performed, following the method established by Nagata and Yamashita [103]. The analysis revealed the presence of chlorophyll a, chlorophyll b, and lycopene in the algal sample. The concentration of chlorophyll a was determined to be 0.58 mg/100 mL, while chlorophyll b was quantified at 0.70 mg/100 mL. Additionally, the pigment lycopene was found at a concentration of 0.16 mg/100 mL. These pigments possess beneficial properties that make them valuable in cosmetic and skincare applications. Chlorophylls have been shown to exhibit antioxidant, anti-inflammatory, and photoprotective effects, contributing to the maintenance of healthy skin. Lycopene, on the other hand, is recognized for its potent antioxidant properties and its potential role in protecting the skin from damage induced by ultraviolet (UV) radiation. The presence of chlorophylls and lycopene in *P. boergesenii* highlights its potential as a natural source of these pigments, which can be utilized in the development of cosmetic formulations aimed at enhancing skin health and providing protection against environmental stressors [104,105].

#### 2.8.3. Quantification of Chlorophylls and Total Carotenoids

The estimation of chlorophylls and total carotenoids in *P. boergesenii* using the acetone–water extraction method yielded interesting results. The concentrations of chlorophyll a and chlorophyll b were determined to be 6.90 µg/mL and 5.44 µg/mL, respectively. These values indicate the presence of significant amounts of chlorophyll pigments in the algal sample. Interestingly, the analysis did not detect any measurable content of total carotenoids in the algal sample. Carotenoids are a diverse group of pigments that contribute to the coloration of plants and algae. They also play vital roles as antioxidants, protecting cells from oxidative damage. While carotenoids are commonly found in algae, their absence in *P. boergesenii* could be attributed to various factors, such as genetic variation, growth conditions, or specific metabolic pathways within the algal species. It is worth noting that the obtained results should be interpreted in the context of the specific extraction method and the sensitivity of the measurement techniques employed. The extraction solvent and wavelength selection for absorbance measurement were based on the method described by Yang et al. [106]. 

When comparing the results, a significant variation can be observed in the pigment content, depending on the extraction method and the solvents used. In general, the chlorophyll content (chlorophyll a, b, and c) shows variations across the different methods. The highest concentrations of chlorophylls were observed in the 100% methanolic extract, followed by the acetone–water mixture (4:1) and the 100% ethanolic extract. The acetone–hexane mixture (2:3) yielded lower concentrations of chlorophylls compared to other methods. Carotenoids, on the other hand, were detected in varying amounts depending on the method used. Carotenoids were present at the highest concentration in the 100% methanolic extract, followed by the 100% ethanolic extract. No carotenoids were detected in the acetone–water mixture (4:1). In terms of specific pigments, the presence of fucoxanthin was not detected in the DMSO–water (4:1) extract. However, phycoerythrin and phycocyanin were detected in significant amounts in the phosphate buffer (pH = 6.8) extract. These variations in pigment content can be attributed to the solubility properties of pigments in different solvents, as well as the selectivity of each solvent in extracting specific pigments. Additionally, factors such as the algal species, growth conditions, and the sensitivity of the measurement techniques employed in each study can contribute to the observed differences. Comparing the results obtained from different methods provides valuable insights into the composition and distribution of pigments in *P. boergesenii*. However, it is important to consider the limitations and specificities of each method and interpret the results in the context of the experimental conditions.

By examining multiple studies and using various extraction methods, a more comprehensive understanding of the pigment composition in *P. boergesenii* can be achieved. This knowledge can have implications for various fields, including ecology, biochemistry, and even applications in industries such as cosmetics and food. The variations in pigment contents highlight the importance of selecting appropriate extraction methods based on the specific pigments of interest. For example, if the focus is on chlorophyll content, the 100% methanol or acetone–water (4:1) extraction methods may be more suitable. On the other hand, for specific pigments like fucoxanthin or phycobiliproteins (phycoerythrin and phycocyanin), different solvent systems or buffer solutions may be necessary to extract and quantify these compounds effectively. Comparing the results from different studies and methods also emphasizes the need for standardization in pigment analysis. Consistency in extraction protocols, solvent selection, and measurement techniques can ensure more reliable and comparable results across different studies. Furthermore, the variations in pigment content can have implications for the potential applications of *P. boergesenii*. Pigments, such as chlorophylls and carotenoids, have antioxidant properties and are known for their potential health benefits. They also contribute to the color and visual appeal of natural products, making them valuable in the cosmetics and food industries. Understanding the pigment composition and contents can aid in the development of products that harness the potential benefits of these pigments.

### 2.9. Analysis of Total Polyphenol Content in P. boergesenii

The obtained result for the total phenol content of *P. boergesenii* was determined to be 86.50 µg/mL. To better understand the significance of this finding, a comparison with other samples or established standards in the field would be beneficial. Unfortunately, such a direct comparison was not conducted in this study, limiting our ability to assess the relative abundance of polyphenols in *P. boergesenii*. However, it is important to note that polyphenols are widely recognized as bioactive compounds found in various plant sources, including marine macroalgae. These compounds have been extensively studied and associated with numerous health-promoting effects, particularly antioxidant and anti-aging properties. Given their antioxidant activity, polyphenols play a vital role in protecting the skin from oxidative stress, thereby contributing to its overall health and appearance. Although a direct comparison was not performed in this study, previous research conducted by Souza et al. [107] and Kim et al. [108] has investigated the polyphenol contents of various seaweed species and reported significant levels of polyphenols. These findings highlight the potential of seaweeds as natural sources of bioactive compounds, including polyphenols. Therefore, the considerable polyphenol content (86.50 µg/mL) observed in *P. boergesenii* suggests its potential as a valuable natural ingredient for cosmetic formulations aiming to provide antioxidant and anti-aging benefits.

The total polyphenol content of *P. boergesenii* was determined using a spectrophotometric method based on the Folin–Ciocâlteu assay. This well-established method allows for accurate quantification of polyphenols and is commonly employed in research. By constructing a standard curve using gallic acid as a reference standard, the concentration of polyphenols in the algal sample was measured. This approach ensures reliable measurement and enables comparisons across studies. In conclusion, the high polyphenol content and the associated antioxidant properties of *P. boergesenii* make it a promising candidate for use in cosmetic applications. However, further research is needed to explore its specific bioactive components and evaluate its efficacy in skincare formulations.

### 2.10. Estimation of Total Protein Content in P. boergesenii 

The total protein content of the *P. boergesenii* sample was determined using the Bradford method, which enables the quantification of protein concentration. In this study, the analysis revealed a total protein content of 113.72 µg/mL in the *P. boergesenii* sample (curve attached in Sheet S2 of the Supplementary Materials). While specific studies focusing on the protein content of *P. boergesenii* are limited, there have been investigations into the protein composition of various marine macroalgae species, providing insights into their potential bioactivity. For instance, Farabegoli et al. [109] conducted a study exploring the protein profiles of different marine macroalgae through gel electrophoresis and proteomic analysis. The researchers identified several protein bands with different molecular weights, indicating the presence of diverse proteins in the studied macroalgal species. These findings suggest that marine macroalgae, including *P. boergesenii*, contain a complex mixture of proteins that may contribute to their potential bioactivity. Furthermore, Dixit et al. [110] examined the protein contents and amino acid compositions of various seaweed species. The researchers reported significant protein contents in the analyzed seaweed samples and observed variations in amino acid composition among the species. These findings highlight the nutritional and functional potential of seaweed proteins. While it is important to note that the exact protein content and composition may vary between different algae species, the presence of proteins in *P. boergesenii* aligns with the general characteristics of marine macroalgae. However, it is necessary to conduct further research to investigate the specific bioactive compounds within *P. boergesenii* and establish their functional properties.

The present study did not directly assess the influence of place of collection, time, and other factors; it is important to recognize their potential impact on the observed differences in data. Variations in chemical composition and bioactivity can arise due to differences in environmental conditions, geographical location, seasonal variations, and other ecological factors. Further research considering these variables can provide a more comprehensive understanding of the relationship between the environment and the properties of marine algae.

### 2.11. Antioxidant Analysis of P. boergesenii Extracts Using the DPPH Method

The results of the DPPH assay demonstrated significant antioxidant activity in both the ethanolic and methanolic extracts of *P. boergesenii* (curves attached in Sheet S3 of the Supplementary Materials). The ethanolic extracts exhibited inhibition percentages ranging from 35.5% at 100 μg/mL to 76.75% at 500 μg/mL, while the methanolic extracts showed inhibition percentages ranging from 46.41% at 100 μg/mL to 73.85% at 500 μg/mL. The IC_50_ values, representing the concentration required to achieve 50% inhibition, were calculated as 36.75 μg/mL for the ethanolic extract and 42.784 μg/mL for the methanolic extract of *P. boergesenii*. Ascorbic acid, used as a reference standard, exhibited IC_50_ values of 9.87 μg/mL for the ethanolic extract and 11.98 μg/mL for the methanolic extract. These specific data demonstrate the substantial antioxidant activity of both the ethanolic and methanolic extracts of *P. boergesenii*, albeit with slightly higher IC_50_ values compared to ascorbic acid. Numerous studies have investigated the antioxidant potential of marine macroalgae, highlighting their significance as rich sources of bioactive compounds with applications in various industries, including cosmetics. Wang, Jonsdottir, and Ólafsdóttir [111] explored the antioxidant activity of different marine macroalgae species and reported substantial scavenging activity against DPPH radicals, which was attributed to the presence of phenolic compounds, carotenoids, and flavonoids with known antioxidant properties. This supports the idea that the antioxidant activity observed in *P. boergesenii* may be due to the presence of similar bioactive compounds.

In another study by El-Shafei, Hegazy, and Acharya [112], various marine macroalgal extracts were evaluated for their antioxidant potential using the DPPH assay, demonstrating significant scavenging activity. This further emphasizes the importance of marine macroalgae as promising sources of natural antioxidants. Similarly, Monteiro et al. [113] investigated the antioxidant activity of different extracts obtained from marine macroalgae, including methanol and ethanol extracts, and observed considerable antioxidant potential. These studies validate and complement our findings, highlighting the significant antioxidant activity exhibited by marine macroalgae, including *P. boergesenii*. The presence of bioactive compounds such as phenolic compounds, carotenoids, and flavonoids in marine macroalgae likely contributes to their antioxidant potential. Based on these scientific findings, the results of our study support the notion that *P. boergesenii* extracts can serve as valuable sources of natural antioxidants for various applications, including cosmetic formulations. The antioxidant activity demonstrated by *P. boergesenii* aligns with the recognized bioactivity of marine macroalgae, validating their potential as a source of natural antioxidants for skincare and cosmetics.

### 2.12. Assessment of Tyrosinase Inhibition Activity of P. boergesenii Extracts

Based on the results obtained from the assay, the *P. boergesenii* extract exhibited a tyrosinase inhibition activity of 62.14%. Tyrosinase is an enzyme that is crucially involved in melanin production, and inhibiting its activity is desirable for addressing hyperpigmentation concerns in the cosmetics industry. In comparison, the reference standard kojic acid, a well-known tyrosinase inhibitor, demonstrated a higher level of tyrosinase inhibition, at 80.37%. Kojic acid has been extensively studied and widely utilized in various cosmetic formulations due to its potent tyrosinase-inhibitory properties. The findings of this study suggest that the *P. boergesenii* extract possesses significant tyrosinase inhibition activity, although slightly lower than that of kojic acid. This indicates the potential of *P. boergesenii* as a natural alternative for developing cosmetic products targeting hyperpigmentation. However, it is important to note that further investigations are necessary to explore the underlying mechanisms of tyrosinase inhibition by the *P. boergesenii* extract. Understanding the specific compounds and their modes of action responsible for the observed inhibitory activity would contribute to the scientific knowledge and potential optimization of the extract’s efficacy. Moreover, the stability and safety of the *P. boergesenii* extract should be thoroughly assessed before considering its application in cosmetic formulations. Stability studies will help to determine the extract’s shelf life, compatibility with other ingredients, and optimal storage conditions. Safety evaluations, including skin irritation and sensitization tests, as well as long-term toxicological assessments, are essential to ensure the extract’s safety profile for cosmetic use. These additional investigations will provide a more comprehensive understanding of the potential of *P. boergesenii* extracts and their suitability for cosmetic applications.

Several studies have explored the potential of marine macroalgae as a source of tyrosinase inhibitors for various applications, including skincare and cosmetic formulations. Im [114] investigated the tyrosinase-inhibitory activity of various marine macroalgal extracts and reported significant tyrosinase inhibition in several algae species, indicating their potential as natural tyrosinase inhibitors. Consistent with these findings, our study demonstrated notable tyrosinase inhibition activity in the ethanolic and methanolic extracts of *P. boergesenii*. In another study by Jo et al. [115], the researchers evaluated the tyrosinase-inhibitory potential of different marine macroalgal extracts and observed significant tyrosinase inhibition in certain algal extracts. They attributed this activity to the presence of phenolic compounds, flavonoids, and other bioactive constituents. These findings support the hypothesis that *P. boergesenii* extracts may contain similar bioactive compounds responsible for tyrosinase inhibition. Fitton et al. [116] investigated the tyrosinase-inhibitory activity of various marine macroalgae species and discovered significant tyrosinase inhibition in some algal extracts. The study highlighted the importance of exploring marine macroalgae as promising sources of bioactive compounds for cosmetic and skincare applications. These related studies corroborate our findings and underscore the potential of marine macroalgae, including *P. boergesenii*, as a source of tyrosinase inhibitors. The presence of phenolic compounds, flavonoids, and other bioactive constituents in these algal extracts may contribute to their tyrosinase-inhibitory activity. Based on these findings, *P. boergesenii* extracts warrant further investigation for their potential use in cosmetic formulations targeting skin pigmentation disorders and related applications. The presence of bioactive compounds with tyrosinase-inhibitory activity in *P. boergesenii* suggests its potential as a natural ingredient for cosmetic products aimed at managing skin pigmentation issues. Our study, investigating the biochemical profile of *P. boergesenii* and its cosmetic potential, complements the findings of Soleimani et al. [117], who evaluated the ethyl acetate fraction of *P. boergesenii* as a biofilter in sunscreen formulations. While Soleimani et al. [117] focused on the antioxidant activity and stability of the fraction, our study encompassed a broader range of analyses, including characterization of functional groups, identification of beneficial compounds, assessment of antioxidant capacity, and evaluation of tyrosinase inhibition. Together, these studies contribute to a comprehensive understanding of the valuable properties of *P. boergesenii* and its potential as an effective ingredient in cosmetics and skincare products.

## 3. Materials and Methods

### 3.1. Collection, Transportation, and Storage Protocol for the Brown Alga P. boergesenii

**Figure 6:**
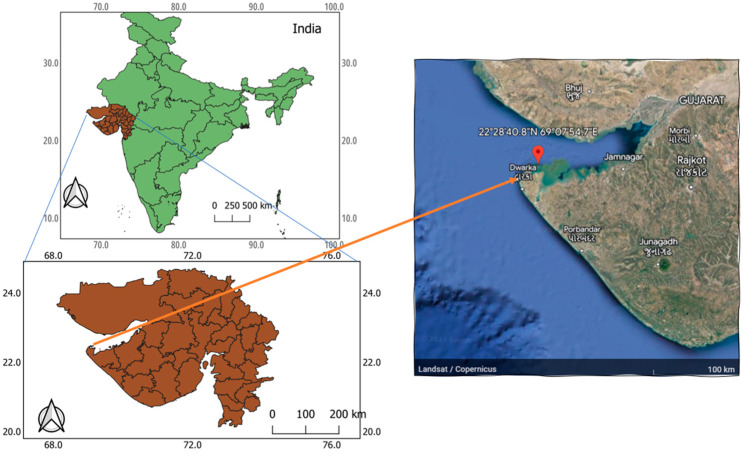
Geographical location of the collection site in the state of Gujarat, India.

**Figure 7:**
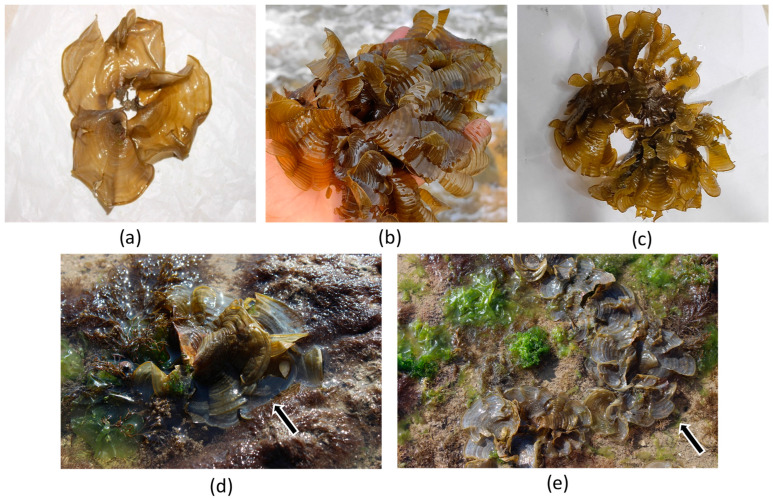
*Padina boergesenii* (Ochrophyta, Phaeophyceae): (**a**–**c**) isolated sample of the macroalga (22°28′40.8″ N 69°07′54.7″ E), (**d**,**e**) onshore view of the *P. boergesenii* in sea water.

The marine macroalgal sample of *Padina boergesenii* was collected from the coordinates “22°28′40.8″ N 69°07′54.7″ E” at the Beyt Dwarka (Bet Dwarka) seacoast, situated on the western coast of Gujarat, India (Figure 6). The algae specimen was identified by Dr. Nilesh H. Joshi, an expert in marine algae identification, who is currently working in the fisheries department at Junagadh Agriculture University, Veraval, Gujarat, India. Ample amounts of the sample were collected (Figure 7) to ensure sufficient material for all characterizations and bioactivity analyses conducted in this study. Upon collection, the samples were immediately submerged in sterile plastic bags filled with seawater to preserve their natural condition during transportation. Careful measures were taken to prevent contamination or damage to the samples. In the laboratory, the samples were stored at 10 °C for 15 days, ensuring the preservation of the natural condition of the sample until further processing. Voucher samples were prepared following standard protocols and deposited for reference, with the assigned voucher number PB-2023-GUJ(02), and stored in the storeroom of the Microbiology Laboratory, Sankalchand Patel University, Visnagar-384315, Gujarat, India. 

For the extraction of bioactive compounds, the collected samples underwent a specific preparation procedure. Initially, the samples were thoroughly washed with deionized water to remove extraneous debris and epiphytes. After washing, the samples were air-dried at room temperature to eliminate excess moisture. Subsequently, the dried samples were finely ground into a powder using a mortar and pestle. The powdered samples of *P. boergesenii* were subjected to extraction using an appropriate solvent system, as detailed in the Methods section. The extraction process involved specific scientific procedures, including washing, drying, grinding, and solvent-based extraction methods, as outlined in the Methods section. Maceration or refluxing methods were employed based on the target bioactive compounds of interest. Following extraction, the solutions were filtered to remove solid particles and impurities. The extracts were then concentrated under reduced pressure using a rotary evaporator. The resulting crude extract was further processed and stored under suitable conditions for subsequent analyses. The collection and preparation of *P. boergesenii* samples were conducted meticulously to ensure sample integrity and provide an adequate quantity for comprehensive characterizations and bioactivity analyses. These preparation and extraction protocols were meticulously followed, adhering to established scientific practices, to ensure the generation of reliable and reproducible results.

### 3.2. Functional Group Analysis of P. boergesenii Using FTIR Spectroscopy

FTIR analysis was carried out in an instrument model 3000 Hyperion Microscope with Vertex 80 FTIR System, Bruker, Germany. 5 mg of shed dried *P. boergesenii* sample was mixed with FT-IR grade Potassium bromide (99% trace metal basis, Sigma-Aldrich, Bengaluru, India) and mixed evenly to obtain a homogenized fine powder. This prepared powder was then kept in a mold and pressed mechanically for 30 s using a sterile spatula to form pellets. This pellet was shifted on the pan and proceeded for analysis. The scanning range of analysis at 400–4000 cm^−1^ wavelength. Based on the peak values in the region of the IR spectrum, the functional groups of the compounds were separated [118,119].

### 3.3. Characterization of Phycocompounds in P. boergesenii by GCMS Analysis

The extract of P. boergesenii was prepared by continuously hot percolating 500 g of finely ground powder obtained from shed-dried algae, using 98% ethanol (Sigma-Aldrich, Bengaluru, India) at 70 °C in a Soxhlet assembly [120]. It was run for 6 h, and then the ethanolic extract *P. boergesenii* was filtered and kept in a dry hot-air oven (RDHO 80, REMI, Bengaluru, India) for 24 h at 40 °C, which was used to evaporate the ethanol (Sigma-Aldrich, Bengaluru, India). The prepared extract was concentrated to dryness in a rotary vacuum evaporator (Sigma Scientific, Chennai, India) under reduced pressure (150 mbar) at 20 °C. The obtained residues of the algal extract were kept separately in airtight vessels and put in a deep freezer maintained at −20 °C (Esquire Biotech, Chennai, India). 

The gas chromatography–mass spectrometry (GCMS) analysis was conducted using a JMS-T100GCV GC model. The selected GC conditions were optimized to achieve efficient separation and characterization of the bioactive compounds present in the extract. A carrier gas, helium (He), was used at a flow rate of 1 mL/min to facilitate the sample’s elution through the column. The injection temperature was set at 200 °C to ensure the vaporization of the sample and its introduction into the column. The column oven temperature was programmed from 50 to 250 °C at a rate of 10 °C/min to achieve proper separation of the compounds. The ionization voltage was maintained at 70 eV, and both the ion source and interface temperatures were set at 250 °C to ensure optimal ionization and transfer of the analytes. The mass range was set from 50 to 600 mass units to cover a wide range of potential compounds. By employing these GC conditions, we obtained gas chromatograms (GCs) and compared the mass spectra of each unknown compound with those of known compounds stored in the NIST (National Institute Standard and Technology) library ver. 2005. Additionally, we calculated the percentage peak area for each compound detected in the selected marine algae samples, providing valuable insights into their relative abundance and distribution [121,122,123].

### 3.4. Comprehensive Profiling of Methanol-Extracted Phycocompounds in P. boergesenii Using GCMS Analysis

The methanolic extraction method was employed to extract phycocompounds from *P. boergesenii*. First, 100 g of shed-dried finely ground powder of *P. boergesenii* was extracted with 1000 mL of 99.8% methanol (Sigma-Aldrich, Bengaluru, India) in a flask for 72 h. The extraction process was carried out twice with fresh methanol solvent to ensure comprehensive extraction. After each extraction, the mixture was filtered, and the supernatants were collected separately. The excess methanol solvent was then removed from the collected supernatants using a rotary vacuum evaporator (Sigma Scientific, Chennai, India) [124,125]. For the subsequent analysis of phycocompounds, the concentrated extracts obtained from the methanolic extraction were subjected to gas chromatography–mass spectrometry (GCMS) analysis. Prior to GCMS analysis, it is important to note that a derivatization step was performed to convert the extracted fatty acids into their methyl ester forms, enhancing their stability and volatility for accurate quantification and identification. The derivatization of fatty acids was carried out using the fatty acid methyl ester (FAME) method, which is a widely accepted and established technique. Briefly, the concentrated extracts were treated with methanol and an acid catalyst, such as sulfuric acid (1% *v*/*v*), to esterify the fatty acids into their methyl ester forms. This step allows for better separation, identification, and quantification of the fatty acids during GCMS analysis. The GCMS analysis was performed using a JMS-T100GCV gas chromatograph coupled with the AccuTof mass detector from JEOL. The column used was an HP5. The ion source temperature and the interface temperature were both maintained at 250 °C. The mass range for the analysis was set from 50 to 600 mass units. During the analysis, helium (He) was used as a carrier gas, with a flow rate of 1 mL/min. The ionization voltage applied was 70 eV. To identify the compounds present in the samples, the obtained gas chromatogram of each algal sample was interpreted, and the mass spectrum of each unknown compound was compared with the known data stored in the NIST library ver. 2005. Additionally, the percentage peak area was calculated for each compound present in the selected marine algae samples.

The choice of methanol as the solvent for fatty acid extraction from *P. boergesenii* was based on established and widely used extraction methods in the scientific literature. The Bligh and Dyer method, described in a classic study by Bligh and Dyer [126], presents a rapid and efficient technique for lipid extraction, where methanol is an essential component of the solvent system. This method has been extensively employed in various lipid extraction studies, including those involving algae. By following this well-established procedure, we aimed to ensure accurate and reliable extraction of fatty acids from *P. boergesenii* for subsequent analysis. The use of methanol as the solvent aligns with established practices in the field and provides a suitable platform for lipid analysis in our research [127,128].

### 3.5. Comprehensive Characterization of Phycocompounds in P. boergesenii Using HRLCMS QTOF

The *P. boergesenii* sample was first dried in a hot-air oven at 40 °C for 24 h; then, this dried alga was ground separately in a mechanical grinder to obtain a uniformly fine powder. One gram of oven-dried *P. boergesenii* sample was kept in a screw-cap tube, and 10 mL of 2 M HCl (hydrochloric acid, Sigma-Aldrich, Bengaluru, India) containing 1% phenol was added. Following this, the tube was closed under N_2_ gas and kept in an electric oven (REMI, Maharashtra, India) at 80 °C for 3 h, before allowing the tube to cool, and its contents were vacuum-filtered through Whatman filter paper grade 41. Following filtration, the filtrate was diluted to 25 mL with ultrapure water in a volumetric flask, separately, and the resulting liquid sample was again membrane-filtered to obtain the hydrolysate. Different types of phycocompounds present in *P. boergesenii* were identified by analyzing samples with the HRLCMS QTOF technique [129,130]. 

The model 6550 iFunnel Q0TODs (Agilent Technologies, Santa Clara, CA, USA) was used, and the scanning range for characterization was set from 150 to 1000 m/z for the MS/MS Dual AJS ESI in ionization mode. The instrumental specifications used were as follows: column: Hypersil GOLD C18; column size: 100 mm × 2.1 mm × 3 µm; gas temperature: 250 °C; gas flow rate: 3 L/min; nebulizer: 35 psig; sheath gas temperature: 300 °C. In addition, the scan parameters included the following: OctopoleRFPeak: 750; nozzle voltage: 1000 V; VCap: 3500; Skimmer1: 65; fragmentor: 175; auxiliary parameters: draw position offset: 0.0 mm; eject speed: 100 µL/min; draw speed: 100 µL/min; vial/well bottom sensing: yes; sample flush-out factor: 5.0; wait time after drawing: 2.0 s.

### 3.6. Quantification of Amino Acids in P. boergesenii Using HRLCMS

For the estimation of amino acids in *P. boergesenii*, 100 mg of shed-dried finely ground powder was weighed separately in different vessels. The weighed *P. boergesenii* sample was then mixed with 12 mL of 6 N HCl (Sigma-Aldrich, Bengaluru, India). The vessels were heat-sealed after filling them with pure nitrogen gas to create an oxygen-free environment. The samples were hydrolyzed by placing the vessels in an electric hot-air oven and maintaining them at a temperature of 120 °C for 16 h. After the hydrolysis process, the samples were carefully removed from the oven and filtered to separate the hydrolyzed mixture from solid residues. Flash evaporation was performed to eliminate any traces of HCl from the samples. To obtain a definite volume of the algal sample residues, a 0.05 N HCl solution was used. The resulting mixture was then filtered through a Whatman filter paper with a pore size of 0.45 µ to remove any particulate matter. The hydrolyzed and filtrated *P. boergesenii* samples were subsequently subjected to analysis using the HRLCMS (high-resolution liquid chromatography–mass spectrometry) method [131,132,133]. For the quantitative calculation of amino acids, the peak areas of the amino acid standards were compared to the peak areas of the corresponding amino acids in the *P. boergesenii* samples. The concentration of each amino acid was determined using an external standard calibration curve. The limit of quantitation (LOQ) value was 10 nmol/mL for the estimated amino acids. For the analysis, the filtrated algae samples were injected into a 6550 iFunnel Q-TOFs instrument (Agilent Technologies, USA) equipped with a Poroshell HPH C18 4.6 × 100 mm, 2.7-micron column. The oven temperature was set to 60 °C, and a non-switching flow method was employed for analysis. Detection was performed in fluorescence mode with post-column derivatization. The instrument specifications provided by Agilent Technologies were followed, including specific settings for the iFunnel Q-TOFs system. To determine the concentration of each amino acid, standards of amino acids were also run, and the results were compared to the standard chromatogram.

### 3.7. Elemental Analysis of P. boergesenii Using the ICP-AES Technique

#### 3.7.1. Sample Preparation and Digestion

For the analysis of different elements, standard analytical pure-grade reagents including nitric acid (HNO_3_), hydrogen peroxide (H_2_O_2_), hydrogen fluoride (HF), and hydrochloric acid (HCl) (Merck, Darmstadt, Germany) were used throughout the experiment. All glassware and plasticware was thoroughly cleaned by soaking overnight in 10% HNO_3_ (Merck, Darmstadt, Germany) and then rinsed with deionized water prior to use. Approximately 0.05 g of *P. boergesenii* was accurately weighed and placed directly into TFM-PTFE (second-generation modified polytetrafluoroethylene) vessels. To each vessel, a separate mixture of reagents consisting of 3 mL of HCl, 1 mL of HNO_3_, 1 mL of HF, and 1 mL of H_2_O_2_ was added. The vessels were quickly sealed to ensure proper digestion [134,135]. Following the addition of the prepared mixtures into the TFM-PTFE vessels, the samples underwent a controlled digestion process using a microwave digester (Titan Microwave system, PerkinElmer, Thane, India). The digestion was performed under the following operational conditions and heating program: (1) ramp time: 130 °C, hold time: 15 min; (2) ramp time: 190 °C, hold time: 15 min. The microwave digester provided precise heating conditions for efficient digestion. After the digestion, the vessels were cooled to 70 °C, vented, and opened. To ensure accurate measurements, the remaining colloids attached to the vessel walls were diluted by adding Milli-Q water to each vessel, resulting in a total volume of 25 mL. Thorough mixing was performed to ensure homogeneity of the contents. Blank samples were also included in the digestion process using the same microwave conditions to account for any background interference. The instrument used had a limit of detection of up to 0.01 ppm, ensuring sensitive detection of the elements present in the samples. The concentrations of elements in the samples were determined by comparing the emission intensities to standard calibration curves generated using certified reference materials.

#### 3.7.2. Element Analysis by ICP-AES

For the analysis of elements using ICP-AES (inductively coupled plasma atomic emission spectrometry), the ARCOS Simultaneous ICP Spectrometer (SPECTRO Analytical Instruments GmbH, Kleve, Germany) equipped with Smart Analyzer Vision 5.01.0921 software and a charge-coupled device (CCD) was employed. The ICP-AES technique allows for the determination of elemental concentrations. All algae samples, including *P. boergesenii*, were analyzed in triplicate to ensure the accuracy and reproducibility of the results.

### 3.8. Quantification of Pigments in P. boergesenii

To assess the cosmetic potential of *P. boergesenii*, a fundamental understanding of its pigment composition is crucial. Pigments are natural compounds found in various organisms, including algae, and are responsible for the vibrant colors observed in these organisms. They play pivotal roles in light absorption, energy transfer, and photoprotection, making them valuable resources for cosmetic applications. By conducting a comprehensive analysis of pigments in *P. boergesenii* using a range of solvents, we aimed to elucidate the specific pigment profiles and concentrations present in this marine alga. The selection of solvents was based on their ability to selectively extract different classes of pigments, as each class possesses unique chemical properties and cosmetic functionalities. Quantification of pigments in triplicate, following established protocols, ensured the reliability and accuracy of the obtained results. This quantitative approach allowed us to determine the concentrations of various pigments, including chlorophylls, carotenoids, fucoxanthin, phycoerythrin, phycocyanin, and lycopene, which are known to exhibit cosmetic benefits.

#### 3.8.1. Quantification of Chlorophylls, Carotenoids, Fucoxanthin, Phycoerythrin, and Phycocyanin

For the extraction of chlorophylls, 1 g of *P. boergesenii* sample was mixed with ≥99.90% purified methanol (34860-SigmaAldrich^®^, Bengaluru, India) and ≥99.8% purified ethanol (34852-M, Sigma-Aldrich^®^, Bengaluru, India). Total carotenoids were extracted using only methanol—not ethanol—while the brown pigment fucoxanthin, commonly found in brown algal species, was extracted using a DMSO–water mixture (4:1, *v*/*v*, Sigma-Aldrich, Bengaluru, India). The red pigments phycoerythrin and phycocyanin were extracted using a phosphate buffer at pH 6.8. Following extraction, the prepared extracts of *P. boergesenii* were subjected to centrifugation to remove any particulate matter. The supernatants were then analyzed for pigment content using a UV–Vis spectrophotometer. The absorbance values were measured at specific wavelengths corresponding to the maximum absorption of each pigment, as indicated in the provided equations. The pigment content was determined using the absorbance values and the formulae based on standard methods. The content of each pigment was expressed as µg/mL of macroalgae [136]. The below set of equations were used to calculate the mentioned pigments:

**Set 1.** Equations for estimation of chlorophyll and carotenoids in 100% methanol.

1. Chl a (μg/mL) = −2.0780 × (A632 − A750) − 6.5079 × (A652 − A750) + 16.2127 × (A665 − A750) − 2.1372 × (A696 − A750)2. Chl b (μg/mL) = −2.9450 × (A632 − A750) − 32.1228 × (A652 − A750) + 13.8255 × (A665 − A750) − 3.0097 × (A696 − A750)3. Chl c (μg/mL) = 34.0115 × (A632 − A750) − 12.7873 × (A652 − A750) + 1.4489 × (A665 − A750) − 2.5812 × (A696 − A750)4. Chl d (μg/mL) = −0.3411 × (A632 − A750) + 0.1129 × (A652 − A750) – 0.2538 × (A665 − A750) + 12.9508 × (A696 − A750)5. Total Chl (μg/mL) = Chl a + Chl b + Chl c + Chl d6. Carotenoids (μg/mL) = 4 × (A480 − A750)100% Ethanol

**Set 2.** Equations for estimation of chlorophyll and carotenoids in 100% ethanol.

1. Chl a (µg/mL) = 0.0604 × (A632 − A750) − 4.5224 × (A649 − A750) + 13.2969 × (A665 − A750) − 1.7453 × (A696 − A750)2. Chl b (µg/mL) = −4.1982 × (A632 − A750) + 25.7205 (A649 − A750) − 7.4096 × (A665 − A750) − 2.7418 × (A696 − A750)3. Chl c (µg/mL) = 28.4593 × (A632 − A750) − 9.9944 (A649 − A750) − 1.9344 × (A665 − A750) − 1.8093 × (A696 − A750)4. Chl d (µg/mL) = −0.2007 × (A632 − A750) + 0.0848 (A649 − A750) − 0.1909 × (A665 − A750) + 12.1302 × (A696 − A750)5. Total Chl (μg/mL) = Chl a + Chl b + Chl c + Chl d

**Set 3.** Equations for estimation of fucoxanthin, phycoerythrin, and phycocyanin.

DMSO: Water (4:1, *v*/*v*)Fucoxanthin (µg/mL) = 7.60 × (A480 − A750) − 5.55 × [(A631 − A750) + (A582 − A750) − 0.297 × (A665 − A750)] − 0.377 × (A665 − A750)Phosphate Buffer (pH = 6.8)1. Phycoerythrin (µg/mL) = [(A565 − A750)/2.41 × 10^6^] × 240,000 × 10^3^2.Phycocyanin (µg/mL) = [(A618 − A750)/1.90 × 10^6^] × 264,000 × 10^3^

#### 3.8.2. Estimation of Chlorophylls and Lycopene

The quantification of chlorophyll a, chlorophyll b, and lycopene followed the standard procedure described by Nagata and Yamashita [103]. Fresh *P. boergesenii* weighing 100 mg was homogenized with a 10 mL acetone–hexane mixture (2:3, Sigma-Aldrich, Bengaluru, India) for 120 s. To maintain sample integrity, the homogenization process was conducted in an ice-water bath. The resulting homogenate was then centrifuged, and the absorbance values were measured at specific wavelengths using a UV–Vis spectrophotometer. The contents of chlorophyll a, chlorophyll b, and lycopene were calculated using the provided equations, and the values were expressed in milligrams per 100 milliliters (mg/100 mL) [103].

**Set 4.** Equations for Chlorophyll a, Chlorophyll b, and Lycopene.

Chlorophyll a (mg/100 mL) = 0.999 × A663 − 0.0989 × A645Chlorophyll b (mg/100 mL) = −0.328 × A663 + 1.77 × A645Lycopene (mg/100 mL) = −0.0485 × A663 + 0.204 × A645 + 0.372 × A505 − 0.0806 × A453A = Absorbance

#### 3.8.3. Estimation of Chlorophylls and Total Carotenoids

The determination of chlorophyll a, chlorophyll b, and total carotenoids in *P. boergesenii* followed the method described by Yang et al. [106]. The extraction process employed an acetone–water mixture (4:1) as the solvent, and the absorbance values were measured at specific wavelengths for each pigment. The contents of these pigments were calculated using the provided equations.

**Set 5.** Equations for chlorophyll a, chlorophyll b, and total carotenoids.

Chlorophyll a (µg/100 mL) = 12.25 × A663.6 − 2.25 × A646.6Chlorophyll b (µg/100 mL) = 20.31 × A646.6 + 4.91 × A663.6Total carotenoids (µg/100 mL) = [1000 × A470 − 2.27(Chl a) – 81.4 (Chl b)]/227A = Absorbance

### 3.9. Analysis of Total Polyphenol Content in P. boergesenii

The spectrophotometric method was used to measure the total polyphenol content in *P. boergesenii*. In this assay, gallic acid was used as a standard (50 µg/mL). First, we added 50 mg of *P. boergesenii* sample separately dissolved in 0.5 mL of dimethyl sulfoxide (DMSO, Sigma-Aldrich, Bengaluru, India), followed by adding demineralized water up to the limit of a 5.0 mL volumetric flask to make a final sample concentration of 1 g/100 mL. Next, we carefully pipetted 0, 0.2, 0.4, 0.6, 0.8, and 1.0 mL of the prepared standard gallic acid solution (50 µg/mL) in each tube and brought all the tubes to a final volume of 1 mL by adding the appropriate amount of distilled water. In the same way, we took different aliquots of the *P. boergesenii* sample and added distilled water to a total volume of 1 mL. Following this, we added 5.0 mL of 1/10 dilution of Folin–Ciocâlteu (FC) reagent (Sigma-Aldrich, Bengaluru, India) to each tube. Afterwards, we added 4.0 mL of a 7.5% sodium carbonate (Na_2_CO_3_) solution to each of the above tubes. Then, these tubes were kept standing at room temperature for 1 h. After one hour of incubation, we read the absorbance values of all of the tubes at 762 nm against deionized water. The concentration of polyphenol in *P. boergesenii* was measured from a standard curve of gallic acid by taking a concentration range from 10 to 50 μg/mL [137].

### 3.10. Estimation of Total Protein Content in P. boergesenii

The total protein content was determined following a standard protocol (https://pubmed.ncbi.nlm.nih.gov/942051/) (accessed on 13 March 2023). To prepare the standard solutions, precise volumes (0, 0.2, 0.4, 0.6, 0.8, and 1.0 mL) of a 100 µg/mL bovine serum albumin (BSA) solution were pipetted into separate labeled test tubes. The total volume of each test tube was adjusted to 1 mL using phosphate buffer. For the *P. boergesenii* samples, different aliquots (0.5, 0.8, 1.0 mL) were taken and adjusted to a total volume of 1 mL to achieve a sample concentration of 1%. To initiate the protein quantification process, 5.0 mL of Bradford reagent was added to each test tube containing the BSA standard solutions and the *P. boergesenii* samples. After the addition of the reagent, thorough mixing was performed for each test tube. Subsequently, the set of tubes was incubated in a dark area for 10 min to allow for color development. Following the incubation period, the absorbance values of the samples were measured at 595 nm using a UV–Vis spectrophotometer, employing a standard method for absorbance measurement. To determine the protein concentration of *P. boergesenii*, a graph was constructed on graph paper by plotting the absorbance values (y-axis) against the concentration of BSA (x-axis). The corresponding protein concentration values for *P. boergesenii* were then determined by plotting the absorbance values of the samples on the standard graph. To obtain the final value of the unknown protein concentration, the protein concentration values were multiplied by the appropriate dilution factor, as necessary, and the average was calculated. This ensured the accuracy and reliability of the protein quantification process [138].

### 3.11. Analysis of P. boergesenii Extracts Using the DPPH Method

The antioxidant activity of methanol and ethanol extracts obtained from *P. boergesenii* was evaluated by assessing their ability to scavenge the stable DPPH (1,1-diphenyl-2-picrylhydrazyl) free radical [139]. To begin, a stock solution of the *P. boergesenii* sample was prepared at a concentration of 1.0 mg/mL and subsequently diluted to final concentrations of 100 μg/mL, 200 μg/mL, 400 μg/mL, 600 μg/mL, and 800 μg/mL. For the analysis, 0.2 mL of each algal solution was individually mixed with 3.8 mL of a 50 μM methanolic solution of DPPH. The resulting mixtures were allowed to react at room temperature for 30 min. Following the incubation period, the absorbance of these mixtures was measured at 517 nm using a UV–Vis spectrophotometer. In order to establish a baseline measurement, a control was promptly measured at 0 min. The inhibition percentage (I%) was calculated using the following formula:

Inhibition percentage (I%) = [(A_control − A_sample)/A_control] × 100

where: A_control represents the absorbance of the control sample, and A_sample refers to the absorbance of the sample or standard.

To further characterize the antioxidant activity of the tested *P. boergesenii* sample, the IC_50_ value was determined. The IC_50_ value represents the concentration at which 50% inhibition is achieved. In order to determine the IC_50_ value, the inhibition percentage (I%) was plotted against the different concentrations of the selected *P. boergesenii* sample. Through this assay, the IC_50_ value was determined, serving as a measure of the antioxidant activity of the tested sample. The calculation and analysis of the IC_50_ value provide valuable insights into the potential effectiveness of *P. boergesenii* as an antioxidant agent.

### 3.12. Assessment of Tyrosinase Inhibition Activity of P. boergesenii Extracts

A modified dopachrome method [140] using L-DOPA as a substrate was employed to assess the tyrosinase inhibition activity of the *P. boergesenii* extract. Kojic acid, a known tyrosinase inhibitor, was used as a reference standard, and both the extracts and kojic acid were dissolved in DMSO at a concentration of 0.1 mg/mL. The assay was conducted using a 96-well microplate, and the absorbance values were measured at 475 nm using an ELISA microplate reader. Each well was labeled appropriately, and 40 μL of the prepared *P. boergesenii* extract in DMSO was transferred to the respective labeled well. Subsequently, 80 μL of phosphate buffer (pH 6.8), 40 μL of tyrosinase enzyme, and 40 μL of L-DOPA were added to each well. A blank sample, which included all of the aforementioned reagents except for L-DOPA, was prepared for comparison with each algae sample. The percentage inhibition of tyrosinase was calculated using the following formula:

% Inhibition = [(A_blank − A_sample)/A_blank] × 100

where: A_blank is the absorbance of the blank sample, and A_sample is the absorbance of the sample or standard. 

## 4. Conclusions

In conclusion, this study presents a comprehensive analysis of *P. boergesenii*, a Phaeophyceae alga, highlighting its potential as a valuable source of bioactive compounds for cosmetic applications. Utilizing various characterization techniques, including GCMS, FTIR, HRLCMS, and ICP-AES, we revealed the diverse biochemical profile and substantial cosmetic benefits of *P. boergesenii*. The alga exhibits a rich composition of active ingredients such as long-chain fatty acids, ergosterol derivatives, and stigmastane derivatives, offering a wide range of skincare advantages such as moisturization, enhanced skin barrier function, antibacterial and antifungal effects, anti-inflammatory properties, and immunostimulatory effects. Additionally, *P. boergesenii* demonstrates skincare benefits through carbohydrate derivatives and other phytochemical compounds, including antioxidant activity, anti-wrinkle effects, acne-fighting properties, moisturizing capabilities, antimicrobial action, and conditioning properties. The presence of amino acids and essential elements further contributes to its skincare advantages, including UV protection, antioxidant activity, inhibition of melanogenesis, skin whitening, anti-aging effects, and collagen synthesis stimulation. Pigment content analysis revealed variations based on the extraction methods and solvents used, impacting the amounts of chlorophylls and carotenoids obtained. Total polyphenol and protein content assessments confirmed the presence of these bioactive compounds, known for their antioxidant properties, in *P. boergesenii*. Notably, ethanolic and methanolic extracts demonstrated significant antioxidant activity and tyrosinase inhibition potential, addressing hyperpigmentation concerns. These findings suggest the diverse cosmetic applications of *P. boergesenii*, including moisturizers, photoprotective formulations, conditioners, skin-whitening products, anti-wrinkle creams, and soothing creams. The extract’s antimicrobial properties and overall contribution to skin health further enhance its appeal. However, to fully unlock the cosmetic potential of *P. boergesenii*, further investigations are warranted. Future studies should focus on elucidating the molecular pathways involved in tyrosinase inhibition and understanding the stability and safety profiles of *P. boergesenii* extracts for cosmetic applications. In vivo evaluations and clinical studies are necessary to assess the efficacy and safety of *P. boergesenii*-based formulations in real-world settings. Exploring the synergistic effects of *P. boergesenii* extracts with other natural compounds or commonly used active ingredients in the cosmetics industry may enhance their efficacy and expand their applications. Moreover, the development of sustainable cultivation methods and extraction techniques will ensure a consistent supply of this valuable marine resource. The utilization of marine-algae-derived compounds, such as *P. boergesenii*, offers a promising avenue in the cosmetics industry due to their effectiveness, lower risks compared to synthetic compounds, and compatibility with the skin’s natural structure. Incorporating *P. boergesenii* extracts into skincare formulations provides consumers with safer and more sustainable alternatives for maintaining skin health and addressing various skincare concerns. 

In conclusion, this study provides valuable insights into the biochemical profile of *P. boergesenii* and its potential as a cosmetic ingredient. The comprehensive analysis revealed the presence of diverse bioactive compounds, including phenolic compounds, fatty acids, peptides, and amino acids, with antioxidant, anti-inflammatory, and skin-nourishing properties. These findings support the notion that *P. boergesenii* holds promise for enhancing skin health in cosmetic formulations. However, further research is warranted to fully substantiate its value and safety as a raw material for cosmetics. Future studies should focus on in-depth formulation development, stability testing, efficacy evaluations, and rigorous safety assessments. Additionally, investigating the synergistic effects of *P. boergesenii* with other cosmetic ingredients may unlock its full potential and broaden its scope of application in various cosmetic products. Continued exploration of *P. boergesenii* in the cosmetics industry will help leverage its valuable properties and contribute to the development of sustainable and effective skincare products.

## Figures and Tables

**Table 1:** Characterization of functional groups in *P. boergesenii* by FTIR analysis.

Frequency (cm^−1^)	Intensity	Band Assignments	Functional Group
604.94	S	C-Cl stretch	Halo compound
	S	C-Br stretch	Halo compound
659.08	S	C-Br stretch	Halo compound
	S	C-Cl stretch	Halo compound
751.88	S	C-Cl stretch	Halo compound
	S	C-H bend	1,2-Disubstituted
	S	C-H bend	Monosubstituted
1038.02	S	S=O stretch	Sulfoxide
	S	C-F stretch	Fluoro compound
	M	C-N stretch	Amine
1099.89	S	C-F starch	Fluoro compound
	S	C-O starch	Aliphatic ether
	S	C-O starch	Secondary alcohol
	M	C-N stretch	Amine
1126.96	S	C-F stretch	Fluoro compound
	S	C-O stretch	Tertiary alcohol
	S	C-O stretch	Aliphatic ether
	M	C-N starch	Amine
1196.56	S	C-F stretch	Fluoro compound
	S	C-O stretch	Tertiary alcohol
	S	C-O stretch	Ester
	M	C-N stretch	Amine
1320.30	S	C-F stretch	Fluoro compound
	S	C-N stretch	Aromatic amine
	S	S=O stretch	Sulfone
	M	O-H bend	Phenol
1343.50	S	C-F Stretch	Fluoro compound
	S	S=O Stretch	Sulfonate
	S	S=O Stretch	Sulfonamide
	S	S=O Stretch	Sulfonic acid
	S	S=O Stretch	Sulfone
	M	O-H bend	Phenol
1521.37	S	N-O stretch	Nitro compound
1648.98	M	C=N Stretch	Imine/Oxime
	M	C=C stretch	Alkene (disubstituted)
	M	C=C stretch	Alkene
	M	C=C stretch	Conjugated alkene
	M	N-H bend	Amine
	M	C=C stretch	Cyclic alkene
	S	C=C Stretch	Alkene (monosubstituted)
1656.71	M	C=C stretch	Alkene (disubstituted)
	M	C=N Stretch	Imine/Oxime
	M	C=C stretch	Alkene (vinyldehyd)
	W	C-H bend	Aromatic compound
1873.25	W	C-H bend	Aromatic compound
2859.29	SB	O-H stretch	Carboxylic acid
	WB	O-H stretch	Alcohol
	SB	N-H Stretch	Amine salt
	M	C-H stretch	Alkane
2925.03	SB	O-H stretch	Carboxylic acid
	WB	O-H stretch	Alcohol
	SB	N-H Stretch	Amine salt
	M	C-H stretch	Alkane
2959.83	SB	O-H stretch	Carboxylic acid
	WB	O-H stretch	Alcohol
	SB	N-H Stretch	Amine salt
	M	C-H stretch	Alkane
3404.51	SB	O-H stretch	Alcohol
3686.79	MS	O-H Stretch	Alcohol

S: strong; M: medium; W: weak; SB: strong broad; SS: strong sharp; WB: weak broad; MS: medium sharp.

**Table 2:** Chemical information of phycocompounds identified in ethanolic extracts of *Padina boergesenii*.

No.	Name of Compound	PubChem ID	Mol. Formula	Mol. Weight (g/mol)	Retention Time (min)	Kovats Index (iu)	% Peak Area
1.	Decane, 6-ethyl-2-methyl-	43923	C_13_H_28_	184.36	12.42	1185	4.32
2.	Cyclopentanol, 2-methyl-, trans-	6432271	C_6_H_12_O	100.16	16.42	849	2.31
3.	Pentadecanoic acid, tripropylsilyl ester	632007	C_24_H_50_O_2_Si	398.7	20.07	2484	1.68
4.	3,7,11,15-Tetramethylhexadec-2-en-1-ol	145386	C_20_H_40_O	296.5	20.77	2045	28.64
5.	Cyclodecanol	15166	C_10_H_20_O	156.26	21.19	1387	2.77
6.	9-(2-Oxiranyl)-1-nonanol	565484	C_11_H_22_O_2_	186.29	21.51	1448	5.33
7.	Phthalic acid,6-ethyl-3-octyl butyl ester	6423866	C_22_H_34_O_4_	362.5	23.23	2505	16.65
8.	Hexadecanoic acid, ethyl ester	12366	C_18_H_36_O_2_	284.5	23.77	1978	6.61
9.	17-Octadecen-1-ol acetate	249903005	C_20_H_38_O_2_	310.5	25.95	2167	5.50
10.	Cyclooctaneacetic acid, 2-oxo-	536995	C_10_H_16_O_3_	184.23	26.79	1647	2.35
11.	Bispalmitic acid 3-methyl-1,2-butanediyl	549812	C_37_H_72_O_4_	581	28.01	3905	0.87
12.	2-Monolinolenin, 2TMS derivative	5362857	C_27_H_52_O_4_Si_2_	496.9	31.55	2804	3.25
13.	Octadecane, 3-ethyl-5-(2-ethylbutyl)-	292285	C_26_H_54_	366.7	34.20	2413	6.97
14.	Benzyl (6Z,9Z,12Z)-6,9,12-octadecatrienoate	5368209	C_25_H_36_O_2_	368.6	34.60	2774	8.54
15.	Oxalic acid, allyl nonyl ester	6420231	C_14_H_24_O_4_	256.34	8.20	1738	4.19

**Table 3:** Chemical information of fatty acids and other bioactive compounds identified in methanolic extracts of *P. boergesenii*.

No.	Name of Compound	PubChem ID	Mol. Formula	Mol. Weight (g/mol)	% Peak Area	Kovats Index (iu)	Retention Time (min)
1	1,2,4-Trioxolane, 3,5-dipropyl-	536099	C_8_H_16_O_3_	160.21	1.05	1086	14.45
2	Phytol	5366244	C_20_H_40_O	296.5	6.01	2045	15.47
3	1,4-Eicosadiene	5365774	C_20_H_38_	278.5	1.63	2007	16.14
4	Hexadecanoic acid, methyl ester	8181	C_17_H_34_O_2_	270.5	2.31	1878	16.82
5	n-Hexadecanoic acid	985	C_16_H_32_O_2_	256.42	17.77	1968	17.49
6	9-Dodecenoic acid, methyl ester, [E]-	5362755	C_13_H_24_O_2_	212.33	1.44	1489	19.66
7	Hexahydrofarnesol	138824	C_15_H_32_O	228.41	2.28	1563	19.90
8	Oleic acid	445639	C_18_H_34_O_2_	282.5	27.85	-	20.39
					38.33	-	11.44
9	Palmitic acid vinyl ester	69658	C_18_H_34_O_2_	282.5	2.18	1968	22.11
10	1,1’-Bicyclopentyl, 2-hexadecyl-	291314	C_26_H_50_	362.7	1	2653	22.83
11	9,17-Octadecadienal, [Z]-	5365667	C_18_H_32_O	264.4	5.92	1997	24.91
					3.13	1997	29.90
12	17-Octadecynoic acid	1449	C_18_H_32_O_2_	280.4	3.34	2165	25.64
13	29-Methylisofucosterol	6443745	C_30_H_50_O	426.7	3.71	2880	27.76
14	Patchouli alcohol	10955174	C_15_H_26_O	222.37	3.14	1420	31.19
15	9-Octadecenal	5283381	C_18_H_34_O	266.5	2.36	2007	34.79

**Table 4:** Chemical information of phycocompounds identified in *P. boergesenii* by the HRLCMS technique.

Phycocompound	Class of Compound	Formula	Retention Time (min)	Mass (Da)	Abundance	Hits (DB)
8-Hydroxy-2chlorodibenzofuran	Organochlorine compound	C_12_H_7_ClO_2_	0.828	218.0135	828077	2
Sulfabenzamide	Sulfur compounds (sulfonamide)	C_13_H_12_N_2_O_3_S	0.829	276.0549	-	4
3-((2-Methyl-3-furyl)sulfanyl)-2-butanone	Aryl sulfide (organosulfur compound)	C_9_H_12_O_2_S	0.897	184.0573	631461	7
8-Amino caprylic acid	Omega-amino fatty acid (carboxylic acids)	C_8_H_17_NO_2_	1.175	159.1249	1110887	6
L-NIO	Amino acids, peptides, and proteins	C_7_H_15_N_3_O_2_	1.272	173.1157	449684	7
Lys Gly	Dipeptide	C_8_H_17_N_3_O_3_	1.318	203.1262	189239	10
Pirbuterol	Amino alcohols (ethanolamines)	C_12_H_20_N_2_O_3_	1.319	240.146	-	10
N-Guanyl histamine	Amines	C_6_H_11_N_5_	1.329	153.1014	143807	6
Pro Pro His	Pro-Pro-His is an oligopeptide.	C_16_H_23_N_5_O_4_	1.894	349.1722	-	10
MeIQx	Quinoxalines (aromatic amine)	C_11_H_11_N_5_	2.375	213.1016	208953	10
Salsolidine	Isoquinolines (organic heterocyclic compound)	C_12_H_17_NO_2_	2.937	207.1253	-	10
Isopentenyladenine-9-glucoside	Organic heterocyclic compound (aminopurine)	C_17_H_25_N_5_O_4_	3.179	363.1882	-	10
2-Amino-1,7,9trimethylimidazo[4,5g]quinoxaline	Heterocyclic compounds, 2-ring (quinoxalines)	C_12_H_13_N_5_	3.408	227.1171	136941	7
Niazirinin	Carbohydrates and carbohydrate derivatives	C_16_H_19_NO_6_	3.831	321.1201	116378	10
Cryptopleurine	Alkaloids	C_24_H_27_NO_3_	3.88	377.2028	92057	10
Beta-butoxyethyl nicotinate	An aromatic carboxylic acid and a member of pyridines	C_12_H_17_NO_3_	3.964	223.12	104913	7
LG 100268	Vitamin B complex (nicotinic acids)	C_24_H_29_NO_2_	4.033	363.2243	177426	7
2-(3’-Methylthio)propyl malic acid	Alcohol	C_8_H_14_O_5_S	4.169	222.0578	92654	10
Gln Phe Lys	Peptide (organic amino compound)	C_20_H_31_N_5_O_5_	4.387	421.2295	146961	10
N-linoleoyl taurine	Fatty amide (fatty acid derivative)	C_20_H_37_NO_4_S	4.6	387.2453	149019	10
Tyr Ile Pro	Peptide	C_20_H_29_N_3_O_5_	4.632	391.2192	268855	10
S-Decyl GSH	Peptide	C_20_H_37_N_3_O_6_S	5.096	447.245	79144	5
ORG 20599	Steroids (pregnanediones)	C_25_H_40_ClNO_3_	5.171	437.2696	6034	10
Nafronyl	Naphthalenes (benzenoid aromatic compound)	C_24_H_33_NO_3_	5.27	383.2521	82233	7
Lys Met Lys	Oligopeptide	C_17_H_35_N_5_O_4_S	5.533	405.2348	156109	7
Benzenemethanol, 2-(2hydroxypropoxy)-3-methyl-	Aromatic ether	C_11_H_16_O_3_	5.68	196.109	719286	10
Acetyl lycopsamine	Pyrrolizines (organic heterocyclic compound)	C_17_H_27_NO_6_	5.791	341.1862	215252	10
Benzenemethanol, 2-(2hydroxypropoxy)-3-methyl-	Aromatic ether	C_11_H_16_O_3_	6.022	196.1092	130269	10
2,6-Dimethoxy-4-(1propenyl)phenol	Phenols	C_11_H_14_O_3_	6.247	194.0938	149475	10
Azuleno(5,6-c)furan-1(3H)-one, 4,4a,5,6,7,7a,8,9-octahydro-3,4,8-trihydroxy-6,6,8-trimethyl-	Terpenoids (sesquiterpenoids)	C_15_H_22_O_5_	6.251	282.1453	113097	10
Hericerin	Amides (lactams)	C_27_H_33_NO_3_	6.316	419.2504	325331	5
Di-n-pentyl phthalate	Phthalate ester (phthalic acids)	C_18_H_26_O_4_	6.811	306.1819	155711	7
N-Formyl-norleucyl-leucylphenylalanyl-methylester	Peptide	C_23_H_35_N_3_O_5_	7.036	433.2662	476213	2
2,4,6-Trimethyl-4-phenyl-1,3 dioxane	Dioxanes	C_13_H_18_O_2_	7.158	206.13	240568	10

**Table 5:** Measurement of different amino acid contents from *Padina boergesenii* by HRLCMS QTOF.

Sr.no	Amino Acids	H2 (nmol/mL)
1	Aspartic acid (Asp)	2034.99
2	Glutamic acid (Glu)	1332.61
3	Asparagine (Asn)	ND
4	Serine (Ser)	768.22
5	Glutamine (Gln)	ND
6	Histidine (His)	ND
7	Glycine (Gly)	1215.22
8	Threonine (Thr)	527.17
9	Arginine (Arg)	498.77
10	Alanine (Ala)	1268.27
11	Tyrosine (Tyr)	233.26
12	Cystine (Cys)	ND
13	Valine (Val)	389.20
14	Methionine (Met)	293.46
15	Norvaline (Nva)	ND
16	Tryptophan (Trp)	ND
17	Phenylalanine (Phe)	392.66
18	Isoleucine (Ile)	244.84
19	Leucine (Leu)	808.49
20	Lysine (Lys)	354.69
21	Hydroxyproline (Hyp)	806.49

ND means less than 10 nmol/mL; calibration curves for each amino acid attached in the Supplementary Materials.

**Table 6:** Mineral content analysis of the selected macroalga by the ICP-AES technique.

Element	(Amount in %)
B	0.013712
Ca	4.099749
Cu	0.000386
Fe	0.244448
K	2.709106
Mg	2.055089
Zn	0.004538
Na	0.073146
Si	20.17574
Se	ND

ND means less than 0.01 ppm.

## Data Availability

Not applicable.

## Supplementary Materials

The following supporting information can be downloaded at: https://www.mdpi.com/article/10.3390/md21070385/s1, Sheet S1: Calibration curves for Amino acid standards; Sheet S2: Calibration curve for total protein content estimation; Sheet S3: Calibration curves for antioxidant analysis by the DPPH method from *Padina boergesenii*.

## References

[1] Kim S.K. (2014). Marine cosmeceuticals. J. Cosmet. Dermatol..

[2] Kerdudo A., Burger P., Merck F., Dingas A., Rolland Y., Michel T., Fernandez X. (2016). Development of a natural ingredient–Natural preservative: A case study. Comptes Rendus Chim..

[3] Hauser R., Calafat A.M. (2005). Phthalates and human health. Occup. Environ. Med..

[4] Garlantézec R., Monfort C., Rouget F., Cordier S. (2009). Maternal occupational exposure to solvents and congenital malformations: A prospective study in the general population. Occup. Environ. Med..

[5] Kalasariya H.S., Yadav V.K., Yadav K.K., Tirth V., Algahtani A., Islam S., Gupta N., Jeon B.H. (2021). Seaweed-based molecules and their potential biological activities: An eco-sustainable cosmetics. Molecules.

[6] Joshi A., Desai A.Y., Mulye V. (2015). Seaweed resources and utilization: An overview. Biotech. Express..

[7] Pereira L., Neto J.M. (2014). Marine Algae: Biodiversity, Taxonomy, Environmental Assessment, and Biotechnology.

[8] Jesumani V., Du H., Aslam M., Pei P., Huang N. (2019). Potential use of seaweed bioactive compounds in skincare—A review. Marine Drugs.

[9] Pradhan B., Bhuyan P.P., Patra S., Nayak R., Behera P.K., Behera C., Behera A.K., Ki J.S., Jena M. (2022). Beneficial effects of seaweeds and seaweed-derived bioactive compounds: Current evidence and future prospective. Biocatal. Agric. Biotechnol..

[10] Venkatesan K., Sivadasan D., Alghazwani Y., Asiri Y.I., Prabahar K., Al-Qahtani A., Mohamed J.M.M., Khan N.A., Krishnaraju K., Paulsamy P. (2023). Potential of seaweed biomass: Snake venom detoxifying action of brown seaweed *Padina boergesenii* against *Naja naja* venom. Biomass Convers. Biorefinery.

[11] Hakim M.M., Patel I.C. (2020). A review on phytoconstituents of marine brown algae. Future J. Pharm. Sci..

[12] John D.M. (2012). Marine Algae (Seaweeds) Associated with Coral Reefs in the Gulf. Coral Reefs of the Gulf: Adaptation to Climatic Extremes.

[13] Guiry M.D. (2013). AlgaeBase. World-Wide Electronic Publication. https://www.algaebase.org.

[14] Abuga K., Nyamweya N. (2021). Alcohol-based hand sanitizers in COVID-19 prevention: A multidimensional perspective. Pharmacy.

[15] Lage R., Mendes C., Zugaib B.M., Abdalla J.A., Costa A. (2017). Cosmeceutical Ingredients: Botanical and Nonbotanical Sources. Dly. Routine Cosmet. Dermatol..

[16] Zhao X., Zhou M. (2022). Review on Chemical Constituents of *Schizonepeta tenuifolia* Briq. and Their Pharmacological Effects. Molecules.

[17] Zaid A.N., Al Ramahi R. (2019). Depigmentation and anti-aging treatment by natural molecules. Curr. Pharm. Des..

[18] Travlou N.A., Giannakoudakis D.A., Algarra M., Labella A.M., Rodríguez-Castellón E., Bandosz T.J. (2018). S-and N-doped carbon quantum dots: Surface chemistry dependent antibacterial activity. Carbon.

[19] Akhtar Z., Ali S.I., Abbas N., Ali M., Khan M.Y., Hasan S.A., Ahmed S., Manzoor S., Lutfi Z. (2020). Evaluation of Antibacterial Potential of New Acid Dyes Based on Substituted Aryl Amines and Amino Hydroxy Sulfonic Acid. J. Chem. Soc. Pak..

[20] Demurtas M., Baldisserotto A., Lampronti I., Moi D., Balboni G., Pacifico S., Vertuani S., Manfredini S., Onnis V. (2019). Indole derivatives as multifunctional drugs: Synthesis and evaluation of antioxidant, photoprotective and antiproliferative activity of indole hydrazones. Bioorganic Chem..

[21] Kim H., Kim J.T., Barua S., Yoo S.Y., Hong S.C., Lee K.B., Lee J. (2018). Seeking better topical delivery technologies of moisturizing agents for enhanced skin moisturization. Expert Opin. Drug Deliv..

[22] Azeem A., Rizwan M., Ahmad F.J., Khan Z.I., Khar R.K., Aqil M., Talegaonkar S. (2008). Emerging role of microemulsions in cosmetics. Recent Pat. Drug Deliv. Formul..

[23] Keng P.S., Basri M., Zakaria M.R.S., Rahman M.A., Ariff A.B., Rahman R.A., Salleh A.B. (2009). Newly synthesized palm esters for cosmetics industry. Ind. Crops Prod..

[24] Hayes D.G. (2017). Fatty acids–based surfactants and their uses. Fatty Acids.

[25] Meckfessel M.H., Brandt S. (2014). The structure, function, and importance of ceramides in skin and their use as therapeutic agents in skin-care products. J. Am. Acad. Dermatol..

[26] Miya G.M., Oriola A.O., Payne B., Cuyler M., Lall N., Oyedeji A.O. (2023). Steroids and Fatty Acid Esters from *Cyperus sexangularis* Leaf and Their Antioxidant, Anti-Inflammatory and Anti-Elastase Properties. Molecules.

[27] Freitas H.R., Ferreira G.D.C., Trevenzoli I.H., Oliveira K.D.J., de Melo Reis R.A. (2017). Fatty acids, antioxidants and physical activity in brain aging. Nutrients.

[28] Bjørklund G., Shanaida M., Lysiuk R., Butnariu M., Peana M., Sarac I., Strus O., Smetanina K., Chirumbolo S. (2022). Natural compounds and products from an anti-aging perspective. Molecules.

[29] Chao C., Génot C., Rodriguez C., Magniez H., Lacourt S., Fievez A., Len C., Pezron I., Luart D., Van Hecke E. (2018). Emollients for cosmetic formulations: Towards relationships between physico-chemical properties and sensory perceptions. Colloids Surf. A Physicochem. Eng. Asp..

[30] Sharmeen J.B., Mahomoodally F.M., Zengin G., Maggi F. (2021). Essential oils as natural sources of fragrance compounds for cosmetics and cosmeceuticals. Molecules.

[31] Dixit D., Reddy C.R.K. (2017). Non-targeted secondary metabolite profile study for deciphering the cosmeceutical potential of red marine macro alga Jania rubens—An LCMS-based approach. Cosmetics.

[32] Taofiq O., Barreiro M.F., Ferreira I.C. (2020). The role of bioactive compounds and other metabolites from mushrooms against skin disorders-a systematic review assessing their cosmeceutical and nutricosmetic outcomes. Curr. Med. Chem..

[33] Jiang T.A. (2019). Health benefits of culinary herbs and spices. J. AOAC Int..

[34] Gad H.A., Roberts A., Hamzi S.H., Gad H.A., Touiss I., Altyar A.E., Kensara O.A., Ashour M.L. (2021). Jojoba Oil: An updated comprehensive review on chemistry, pharmaceutical uses, and toxicity. Polymers.

[35] Yang M., Zhou M., Song L. (2020). A review of fatty acids influencing skin condition. J. Cosmet. Dermatol..

[36] Alander J.T. (2012). Chemical and Physical Properties of Emollients. Treatment of Dry Skin Syndrome: The Art and Science of Moisturizers.

[37] Husein el Hadmed H., Castillo R.F. (2016). Cosmeceuticals: Peptides, proteins, and growth factors. J. Cosmet. Dermatol..

[38] Sarkar R., Podder I., Gokhale N., Jagadeesan S., Garg V.K. (2017). Use of vegetable oils in dermatology: An overview. Int. J. Dermatol..

[39] Kelm G.R., Wickett R.R. (2017). The role of fatty acids in cosmetic technology. Fatty Acids.

[40] Cochran S., Anthonavage M. (2014). Fatty acids, fatty alcohols, synthetic esters and glycerin applications in the cosmetic industry. Lipids and Skin Health.

[41] Yu R.J., Van Scott E.J. (2004). Alpha-hydroxyacids and carboxylic acids. J. Cosmet. Dermatol..

[42] Draelos Z.D. (2018). The science behind skin care: Moisturizers. J. Cosmet. Dermatol..

[43] Silva R.O., Sousa F.B.M., Damasceno S.R., Carvalho N.S., Silva V.G., Oliveira F.R.M., Sousa D.P., Aragão K.S., Barbosa A.L., Freitas R.M. (2014). Phytol, a diterpene alcohol, inhibits the inflammatory response by reducing cytokine production and oxidative stress. Fundam. Clin. Pharmacol..

[44] Sánchez-Marzo N., Pérez-Sánchez A., Barrajón-Catalán E., Castillo J., Herranz-López M., Micol V. (2020). Rosemary diterpenes and flavanone aglycones provide improved genoprotection against uv-induced DNA damage in a human skin cell model. Antioxidants.

[45] Ribeaucourt D., Bissaro B., Lambert F., Lafond M., Berrin J.G. (2022). Biocatalytic oxidation of fatty alcohols into aldehydes for the flavors and fragrances industry. Biotechnol. Adv..

[46] Kang S.Y., Um J.Y., Chung B.Y., Lee S.Y., Park J.S., Kim J.C., Park C.W., Kim H.O. (2022). Moisturizer in patients with inflammatory skin diseases. Medicina.

[47] Ahmad A., Ahsan H. (2020). Lipid-based formulations in cosmeceuticals and biopharmaceuticals. Biomed. Dermatol..

[48] Vaughn A.R., Clark A.K., Sivamani R.K., Shi V.Y. (2018). Natural oils for skin-barrier repair: Ancient compounds now backed by modern science. Am. J. Clin. Dermatol..

[49] Ahmed I.A., Mikail M.A. (2023). Anti-aging skincare: The natural and organic way. Anti-Aging Pharmacology.

[50] Ashawat M., Banchhor M., Saraf S., Saraf S. (2009). Herbal Cosmetics: Trends in Skin Care Formulation. Pharmacogn. Rev..

[51] Dahiya S., Dahiya R. (2022). Potential of colloidal carriers for nanocosmeceutical applications. Nanocosmeceuticals.

[52] Mohiuddin A.K. (2019). Skin care creams: Formulation and use. Dermatol. Clin. Res..

[53] Yu M., Wan S., Song H., Zhang Y., Wang C., Wang H., Wang H. (2021). Sensory-based identification of aroma-active compounds in hotpot seasoning before and after boiling. Molecules.

[54] Salehi B., Quispe C., Sharifi-Rad J., Cruz-Martins N., Nigam M., Mishra A.P., Konovalov D.A., Orobinskaya V., Abu-Reidah I.M., Zam W. (2021). Phytosterols: From preclinical evidence to potential clinical applications. Front. Pharmacol..

[55] Abdelhamed F.M., Abdeltawab N.F., ElRakaiby M.T., Shamma R.N., Moneib N.A. (2022). Antibacterial and anti-inflammatory activities of Thymus vulgaris essential oil nanoemulsion on acne vulgaris. Microorganisms.

[56] Abozeid D., Fawzy G., Issa M., Abdeltawab N., Soliman F. (2023). Medicinal Plants and their Constituents in the Treatment of Acne vulgaris. Biointerface Res. Appl. Chem..

[57] Mistry N. (2017). Guidelines for formulating anti-pollution products. Cosmetics.

[58] Kalasariya H.S., Patel N.B., Yadav A., Perveen K., Yadav V.K., Munshi F.M., Yadav K.K., Alam S., Jung Y.K., Jeon B.H. (2021). Characterization of fatty acids, polysaccharides, amino acids, and minerals in marine macroalga *Chaetomorpha crassa* and evaluation of their potentials in skin cosmetics. Molecules.

[59] Torres A., Rego L., Martins M.S., Ferreira M.S., Cruz M.T., Sousa E., Almeida I.F. (2023). How to Promote Skin Repair? In-Depth Look at Pharmaceutical and Cosmetic Strategies. Pharmaceuticals.

[60] van Smeden J., Janssens M., Kaye E.C., Caspers P.J., Lavrijsen A.P., Vreeken R.J., Bouwstra J.A. (2014). The importance of free fatty acid chain length for the skin barrier function in atopic eczema patients. Exp. Dermatol..

[61] Jaricot M., Malhiac C., Chao C., Merlaud F., Grisel M., Savary G. (2022). Understanding of the residual odour of fatty esters used as emollient in cosmetic products. Int. J. Cosmet. Sci..

[62] Benchagra L., Berrougui H., Islam M.O., Ramchoun M., Boulbaroud S., Hajjaji A., Fulop T., Ferretti G., Khalil A. (2021). Antioxidant effect of moroccan pomegranate (*Punica granatum* L. *sefri* variety) extracts rich in punicalagin against the oxidative stress process. Foods.

[63] Choi D.Y., Lee Y.J., Hong J.T., Lee H.J. (2012). Antioxidant properties of natural polyphenols and their therapeutic potentials for Alzheimer’s disease. Brain Res. Bull..

[64] Sethi A., Kaur T., Malhotra S.K., Gambhir M.L. (2016). Moisturizers: The slippery road. Indian J. Dermatol..

[65] Nilforoushzadeh M.A., Amirkhani M.A., Zarrintaj P., Salehi Moghaddam A., Mehrabi T., Alavi S., Mollapour Sisakht M. (2018). Skin care and rejuvenation by cosmeceutical facial mask. J. Cosmet. Dermatol..

[66] Schagen S.K. (2017). Topical peptide treatments with effective anti-aging results. Cosmetics.

[67] Ferreira M.S., Magalhães M.C., Sousa-Lobo J.M., Almeida I.F. (2020). Trending anti-aging peptides. Cosmetics.

[68] Shanbhag S., Nayak A., Narayan R., Nayak U.Y. (2019). Anti-aging and sunscreens: Paradigm shift in cosmetics. Adv. Pharm. Bull..

[69] Yadav A.R., Mohite S.K. (2020). Potential role of peptides for development of cosmeceutical skin products. Res. J. Top. Cosmet. Sci..

[70] Kim D.U., Chung H.C., Choi J., Sakai Y., Lee B.Y. (2018). Oral intake of low-molecular-weight collagen peptide improves hydration, elasticity, and wrinkling in human skin: A randomized, double-blind, placebo-controlled study. Nutrients.

[71] Aburjai T., Natsheh F.M. (2003). Plants used in cosmetics. Phytother. Res. Int. J. Devoted Pharmacol. Toxicol. Eval. Nat. Prod. Deriv..

[72] Eldeen I.M.S., Elgorashi E.E., Van Staden J. (2005). Antibacterial, anti-inflammatory, anti-cholinesterase and mutagenic effects of extracts obtained from some trees used in South African traditional medicine. J. Ethnopharmacol..

[73] Rembe J.D., Fromm-Dornieden C., Stuermer E.K. (2018). Effects of vitamin B complex and vitamin C on human skin cells: Is the perceived effect measurable?. Adv. Ski. Wound Care.

[74] Gupta A.D., Rajpurohit D. (2011). Antioxidant and antimicrobial activity of nutmeg (*Myristica fragrans*). Nuts and Seeds in Health and Disease Prevention.

[75] Thapa N., Thapa P., Bhandari J., Niraula P., Shrestha N., Shrestha B.G. (2016). Study of phytochemical, antioxidant and antimicrobial activity of Artocarpus heterophyllus. Nepal J. Biotechnol..

[76] Leandro A., Pereira L., Gonçalves A. (2019). Diverse Applications of Marine Macroalgae. Mar. Drugs..

[77] Corsetti G., D’Antona G., Dioguardi F.S., Rezzani R. (2010). Topical application of dressing with amino acids improves cutaneous wound healing in aged rats. Acta Histochem..

[78] Veis A., Anesey J. (1965). Modes of intermolecular cross-linking in mature insoluble collagen. J. Biol. Chem..

[79] Choi H.-R., Kang Y.-A., Ryoo S.-J., Shin J.-W., Na J.-I., Huh C.-H., Park K.-C. (2012). Stem cell recovering effect of copper-free GHK in skin. J. Pept. Sci..

[80] Murakami H., Shimbo K., Inoue Y., Takino Y., Kobayashi H. (2011). Importance of amino acid composition to improve skin collagen protein synthesis rates in UV-irradiated mice. Amino Acids..

[81] Kawashima M., Yokose U., Hachiya A., Fujimura T., Tsukahara K., Kawada H., Kitahara T., Takema Y., Terui T., Nakagawa H. (2013). Improvement of crow’s feet lines by topical application of 1-carbamimidoyl-L-proline (CLP). Eur. J. Dermatol..

[82] Yamane T., Morioka Y., Kitaura Y., Iwatsuki K., Shimomura Y., Oishi Y. (2018). Branched-chain amino acids regulate type I tropocollagen and type III tropocollagen syntheses via modulation of mTOR in the skin. Biosci. Biotechnol. Biochem..

[83] Puviani M., Agostinis F., Milani M. (2014). Barrier repair therapy for facial atopic eczema with a non-steroidal emollient cream containing rhamnosoft, ceramides and iso-leucine. A six-case report series. Minerva Pediatr..

[84] Brenner M., Hearing V.J. (2008). The Protective Role of Melanin Against UV Damage in Human Skin. Photochem. Photobiol..

[85] Serre C., Busuttil V., Botto J.-M. (2018). Intrinsic and extrinsic regulation of human skin melanogenesis and pigmentation. Int. J. Cosmet. Sci..

[86] Sardana K., Garg V.K. (2010). An observational study of methionine-bound zinc with antioxidants for mild to moderate acne vulgaris. Dermatol. Ther..

[87] Wu G., Bazer F.W., Burghardt R.C., Johnson G.A., Kim S.W., Knabe D.A., Li P., Li X., McKnight J.R., Satterfield M.C. (2010). Proline and hydroxyproline metabolism: Implications for animal and human nutrition. Amino Acids..

[88] Elias P.M., Ahn S.K., Denda M., Brown B.E., Crumrine D., Kimutai L.K., Kömüves L., Lee S.H., Feingold K.R. (2002). Modulations in Epidermal Calcium Regulate the Expression of Differentiation-Specific Markers. J. Investig. Dermatol..

[89] Matz H., Orion E., Wolf R. (2003). Balneotherapy in dermatology. Dermatol. Ther..

[90] Denda M., Katagiri C., Hirao T., Maruyama N., Takahashi M. (1999). Some magnesium salts and a mixture of magnesium and calcium salts accelerate skin barrier recovery. Arch. Dermatol. Res..

[91] Schempp C.M., Dittmar H.C., Hummler D., Simon-Haarhaus B., Schöpf E., Simon J.C., Schulte-Mönting J. (2000). Magnesium ions inhibit the antigen-presenting function of human epidermal Langerhans cells *in vivo* and *in vitro*. Involvement of ATPase, HLA-DR, B7 molecules, and cytokines. J. Investig. Dermatol..

[92] Food and Drug Administration, HHS (2003). Skin protectant drug products for over-the-counter human use; final monograph. Final rule. Fed. Regist..

[93] Food and Drug Administration (2019). Sunscreen Drug Products for Over-the-Counter Human Use. Amendment to the Tentative Final Monograph; Enforcement Policy. Fed. Regist..

[94] Higdon J., Drake V.J. (2012). An Evidenced-Based Approach to Vitamins and Minerals.

[95] Antoniou C., Stefanaki C. (2006). Cosmetic camouflage. J. Cosmet. Dermatol..

[96] Pickart L. (2008). The human tri-peptide GHK and tissue remodeling. J. Biomater. Sci. Polym. Ed..

[97] Fowler Jr J.F., Woolery-Lloyd H., Waldorf H., Saini R. (2010). Innovations in natural ingredients and their use in skin care. J. Drugs Dermatol. JDD.

[98] Leenutaphong V. (1995). Relationship between skin color and cutaneous response to ultraviolet radiation in Thai. Photodermatol. Photoimmunol. Photomed..

[99] O’Connor I., O’Brien N. (1998). Modulation of UVA light-induced oxidative stress by β-carotene, lutein and astaxanthin in cultured fibroblasts. J. Dermatol. Sci..

[100] Wang H.M.D., Chen C.C., Huynh P., Chang J.S. (2015). Exploring the potential of using algae in cosmetics. Bioresour. Technol..

[101] Alves A.L., Marques A.L., Martins E., Silva T.H., Reis R.L. (2017). Cosmetic potential of marine fish skin collagen. Cosmetics.

[102] Zhang H., Tang Y., Zhang Y., Zhang S., Qu J., Wang X., Kong R., Han C., Liu Z. (2015). Fucoxanthin: A promising medicinal and nutritional ingredient. Evid. Based Complement. Altern. Med..

[103] Nagata M., Yamashita I. (1992). Simple method for simultaneous determination of chlorophyll and carotenoids in tomato fruit. Nippon. Shokuhin Kogyo Gakkaishi.

[104] Kadam S.U., Tiwari B.K., O’Donnell C.P. (2013). Application of novel extraction technologies for bioactives from marine algae. J. Agric. Food Chem..

[105] Khoo H.E., Prasad K.N., Kong K.W., Jiang Y., Ismail A. (2011). Carotenoids and their isomers: Color pigments in fruits and vegetables. Molecules.

[106] Yang C.M., Chang K.W., Yin M.H., Huang H.M. (1998). Methods for the determination of the chlorophylls and their derivatives. Taiwania.

[107] Souza B.W., Cerqueira M.A., Bourbon A.I., Pinheiro A.C., Martins J.T., Teixeira J.A., Coimbra M.A., Vicente A.A. (2012). Chemical characterization and antioxidant activity of sulfated polysaccharide from the red seaweed *Gracilaria birdiae*. Food Hydrocoll..

[108] Kim K.N., Heo S.J., Kang S.M., Ahn G., Jeon Y.J. (2010). Fucoxanthin induces apoptosis in human leukemia HL-60 cells through a ROS-mediated Bcl-xL pathway. Toxicol. Vitr..

[109] Farabegoli F., Santaclara F.J., Costas D., Alonso M., Abril A.G., Espiñeira M., Ortea I., Costas C. (2023). Exploring the Anti-Inflammatory Effect of Inulin by Integrating Transcriptomic and Proteomic Analyses in a Murine Macrophage Cell Model. Nutrients.

[110] Dixit D.C., Reddy C.R.K., Balar N., Suthar P., Gajaria T., Gadhavi D.K. (2018). Assessment of the nutritive, biochemical, antioxidant and antibacterial potential of eight tropical macro algae along Kachchh coast, India as human food supplements. J. Aquat. Food Prod. Technol..

[111] Wang T., Jonsdottir R., Ólafsdóttir G. (2009). Total phenolic compounds, radical scavenging and metal chelation of extracts from Icelandic seaweeds. Food Chem..

[112] El-Shafei R., Hegazy H., Acharya B. (2021). A review of antiviral and antioxidant activity of bioactive metabolite of macroalgae within an optimized extraction method. Energies.

[113] Monteiro M., Santos R.A., Iglesias P., Couto A., Serra C.R., Gouvinhas I., Barros A., Oliva-Teles A., Enes P., Díaz-Rosales P. (2020). Effect of extraction method and solvent system on the phenolic content and antioxidant activity of selected macro-and microalgae extracts. J. Appl. Phycol..

[114] Im D.Y. (2014). Antioxidative activity and tyrosinase inhibitory activity of the extract and fractions from *Arctium lappa* roots and analysis of phenolic compounds. Korean J. Pharmacogn..

[115] Jo J.H., Kim D., Lee S., Lee T.K. (2005). Total phenolic contents and biological activities of Korean seaweed extracts. Food Sci. Biotechnol..

[116] Fitton J.H., Dell’Acqua G., Gardiner V.A., Karpiniec S.S., Stringer D.N., Davis E. (2015). Topical benefits of two fucoidan-rich extracts from marine macroalgae. Cosmetics.

[117] Soleimani S., Yousefzadi M., Nezhad S.B.M., Pozharitskaya O.N., Shikov A.N. (2023). Utilization of the ethyl acetate fraction of *Padina boergesenii* as a natural UV filter in sunscreen cream formulation. Life.

[118] Moubayed N.M., Al Houri H.J., Al Khulaifi M.M., Al Farraj D.A. (2017). Antimicrobial, antioxidant properties and chemical composition of seaweeds collected from Saudi Arabia (Red Sea and Arabian Gulf). Saudi J. Biol. Sci..

[119] Suman T.Y., Rajasree S.R., Kirubagaran R. (2015). Evaluation of zinc oxide nanoparticles toxicity on marine algae *Chlorella vulgaris* through flow cytometric, cytotoxicity and oxidative stress analysis. Ecotoxicol. Environ. Saf..

[120] Ciolino L.A., Ranieri T.L., Taylor A.M. (2018). Commercial cannabis consumer products part 1: GC–MS qualitative analysis of cannabis cannabinoids. Forensic Sci. Int..

[121] Kavitha J., Palani S. (2016). Phytochemical screening, GC-MS analysis and antioxidant activity of marine algae *Chlorococcum humicola*. World J. Pharm. Pharm. Sci..

[122] Tatipamula V.B., Killari K.N., Prasad K., Rao G.S.N.K., Talluri M.R., Vantaku S., Bilakanti D., Srilakshmi N. (2019). Cytotoxicity studies of the chemical constituents from marine algae *Chara baltica*. Indian J. Pharm. Sci..

[123] Mustapa A.N., Martin Á., Mato R.B., Cocero M.J. (2015). Extraction of phytocompounds from the medicinal plant *Clinacanthus nutans* Lindau by microwave-assisted extraction and supercritical carbon dioxide extraction. Ind. Crops Prod..

[124] Ragunathan V., Pandurangan J., Ramakrishnan T. (2019). Gas chromatography-mass spectrometry analysis of methanol extracts from marine red seaweed *Gracilaria corticata*. Pharmacogn. J..

[125] Cyriac B., Eswaran K. (2015). GC-MS determination of bioactive components of *Gracilaria dura* (C. Agardh) J. Agardh. Sci. Res. Rep..

[126] Bligh E.G., Dyer W.J. (1959). A rapid method of total lipid extraction and purification. Can. J. Biochem. Physiol..

[127] Cavonius L.R., Carlsson N.G., Undeland I. (2014). Quantification of total fatty acids in microalgae: Comparison of extraction and transesterification methods. Anal. Bioanal. Chem..

[128] Liu Y., Liu Y., Jiao D., Lu C., Lou Y., Li N., Wang G., Wang H. (2021). Synthesis and release of fatty acids under the interaction of Ulva pertusa and Heterosigma akashiwo by stable isotope analysis. Ecotoxicol. Environ. Saf..

[129] Salunke M., Wakure B., Wakte P. (2022). HR-LCMS assisted phytochemical screening and an assessment of anticancer activity of *Sargassum Squarrossum* and *Dictyota Dichotoma* using *in vitro* and molecular docking approaches. J. Mol. Struct..

[130] Salunke M.A., Wakure B.S., Wakte P.S. (2022). High-resolution liquid chromatography and mass spectrometry (HR-LCMS) assisted phytochemical profiling and an assessment of anticancer activities of *Gracilaria foliifera* and *Turbinaria conoides* using in vitro and molecular docking analysis. J. Biomol. Struct. Dyn..

[131] Rosic N.N., Braun C., Kvaskoff D. (2015). Extraction and analysis of mycosporine-like amino acids in marine algae. Nat. Prod. Mar. Algae Methods Protoc..

[132] Javith M.A., Balange A.K., Xavier M., Hassan M.A., Sanath Kumar H., Nayak B.B., Krishna G. (2022). Comparative studies on the chemical composition of inland saline reared *Litopenaeus vannamei*. J. Culin. Sci. Technol..

[133] Carreto J.I., Carignan M.O., Montoya N.G. (2005). A high-resolution reverse-phase liquid chromatography method for the analysis of mycosporine-like amino acids (MAAs) in marine organisms. Mar. Biol..

[134] Falandysz J., Szymczyk K., Ichihashi H., Bielawski L., Gucia M., Frankowska A., Yamasaki S.I. (2001). ICP/MS and ICP/AES elemental analysis (38 elements) of edible wild mushrooms growing in Poland. Food Addit. Contam..

[135] Murugaiyan K., Narasimman S. (2012). Elemental composition of *Sargassum longifolium* and *Turbinaria conoides* from Pamban Coast, Tamilnadu. Int. J. Res. Biol. Sci..

[136] Osório C., Machado S., Peixoto J., Bessada S., Pimentel F.B., Alves R.C., Oliveira M.B.P. (2020). Pigments content (chlorophylls, fucoxanthin and phycobiliproteins) of different commercial dried algae. Separations.

[137] Wijaya C., Elya B., Yanuar A. (2018). Study of tyrosinase inhibitory activity and phytochemical screening of Cassia Fistula L. Leaves. Int. J. Appl. Pharm..

[138] Bradford M.M. (1976). A rapid and sensitive method for the quantitation of microgram quantities of protein utilizing the principle of protein-dye binding. Anal. Biochem..

[139] Maheswari M.U., Reena A., Sivaraj C. (2017). GC-MS analysis, antioxidant and antibacterial activity of the brown algae, Padina tetrastromatica. Int. J. Pharm. Sci. Res..

[140] Salleh W.M.N.H.W., Ahmad F., Yen K.H. (2014). Antioxidant and anti-tyrosinase activities from Piper officinarum C. DC (Piperaceae). J. Appl. Pharm. Sci..

